# Field traffic-induced soil compaction under moderate machine-field conditions affects soil properties and maize yield on sandy loam soil

**DOI:** 10.3389/fpls.2023.1002943

**Published:** 2023-06-20

**Authors:** Muhammad Mohsin Nawaz, Mehmood Ali Noor, Hojatollah Latifmanesh, Xinbing Wang, Wei Ma, Weijian Zhang

**Affiliations:** ^1^ Institute of Crop Sciences, Chinese Academy of Agricultural Sciences, Key Laboratory of Crop Physiology & Ecology, Ministry of Agriculture, Beijing, China; ^2^ Department of Agroecology - Soil Physics and Hydropedology, Aarhus University, Tjele, Denmark; ^3^ Institute of Environmental and Agricultural Sciences, Faculty of Life Sciences, University of Okara, Okara, Punjab, Pakistan; ^4^ Department of Agronomy and Plant Breeding, Faculty of Agriculture, Yasouj University, Yasouj, Iran

**Keywords:** field traffic, soil compaction, root distribution, penetration resistance, maize yield

## Abstract

Soil compaction due to field trafficking involves a complex interplay of machine-soil properties. In contrast to previous studies simulating worst field scenarios, this two-year field experiment investigated the effects of traffic-induced compaction involving moderate machine operational specifications (axle load, 3.16 Mg; mean ground contact pressure, 77.5 kPa) and lower field moisture contents (< field capacity) at the time of trafficking on soil physical properties, spatial root distribution, and corresponding maize growth and grain yield in sandy loam soil. Two compaction levels, i.e. two (C2) and six (C6) vehicle passes, were compared with a control (C0). Two maize (*Zea mays* L.) cultivars, i.e. ZD-958 and XY-335, were used. Results showed topsoil (< 30 cm) compaction with increases in bulk density (BD) and penetration resistance (PR) up to 16.42% and 127.76%, respectively, in the 10-20 cm soil layer in 2017. Field trafficking resulted in a shallower and stronger hardpan. An increased number of traffic passes (C6) aggravated the effects, and the carryover effect was found. Higher BD and PR impaired root proliferation in deeper layers of topsoil (10-30 cm) and promoted shallow horizontal root distribution. However, XY-335, compared with ZD-958, showed deeper root distribution under compaction. Compaction-induced reductions in root biomass and length densities were respectively up to 41% and 36% in 10-20 cm and 58% and 42% in the 20-30 cm soil layer. Consequent yield penalties (7.6%-15.5%) underscore the detriments of compaction, even only in topsoil. In crux, despite their low magnitude, the negative impacts of field trafficking under moderate machine-field conditions after just two years of annual trafficking foreground the challenge of soil compaction.

## 1 Introduction

Soil compaction is a component of soil degradation ‘syndrome’ (Batey, 2009), which plays a role as a causal agent in many soil-related problems such as soil erosion, nutrient depletion, pollution (Hartemink, 2008), and greenhouse gas emissions (Gregorich et al., 2014). Vehicular traffic, a major cause of soil compaction on arable lands, has been revolutionized in past decades. The size and weight of agricultural machinery have been tremendously increased, aggravating the problem of soil compaction by increasing the severity and depth of compacted zone (Chamen et al., 2015; Sivarajan et al., 2018). Most previous studies targeted or simulated highly vulnerable or worst-case scenarios. However, very few studies focused on moderate scenarios such as moderate operational characteristics of machines and relatively better field conditions at the time of trafficking.

The location and extent of traffic-induced compaction are results of a complex interplay of intrinsic soil properties, field conditions under which trafficking takes place, and the specifications of employed machinery. Generally, subsoil compaction is controlled by axle load, whereas topsoil compaction is governed by ground contact pressure (Botta et al., 1999 and Duiker, 2004b; Botta et al., 2008). The other two important factors that govern the extent of compaction are soil properties (such as texture, organic matter, mechanical strength, and tilth (de Lima et al., 2017)) and soil moisture at the time of wheeling (Hamza and Anderson, 2005; Batey, 2009). Studies simulating worst conditions for compaction usually apply traffic at moisture contents greater than or near field capacity (Sidhu and Duiker, 2006). While trafficking in highly moist field conditions might be ineluctable under certain cropping systems or regions, it is not necessarily the case in most cereal cropping systems in arid and semi-arid regions.

Huang-Huai-Hai (HHH) region, where summer maize and winter wheat are the dominant crops, accounts for almost 70% of the total maize cropping area in North China (Meng et al., 2006). Common tillage practices of rotary (in wheat) and reduced tillage (in maize) and increased farm mechanization in this region have posed a serious threat of traffic-induced compaction (Feike et al., 2012; Zhai et al., 2017). The average plough pan depth in the HHH region is just 17.2 cm, which is almost the same as China’s national average of 16.5 cm but much shallower compared to 35 cm in the USA. Previously, compaction has been investigated more in Northeast China (Cai et al., 2014) where soils are mostly Mollisols; but relatively less information is available regarding HHH despite being prone to more field trafficking due to the double cropping system.

Reduced root growth and altered root distribution due to compaction severely hinder the effective utilization of nutrients and moisture (Bengough et al., 2006; Schjønning et al., 2016a). Consequently, the growth and functions of the aboveground plants are adversely affected effectuating yield reductions (Tubeileh et al., 2003; Shah et al., 2017). However, a vast variation among results exists. A study in Europe observed that compaction-induced yield penalties ranged from approximately half the production of uncompacted land to positive effects (Hallett et al., 2012). Similarly, while compaction-induced yield penalties of up to 43% in maize are reported (Voorhees, 2000), some studies suggested no significant differences in maize yield due to increased traffic (Gelder et al., 2007; Sivarajan et al., 2018). These conflicting findings are mainly due to the complexity and multi-disciplinary nature of the compaction problem, where prevailing conditions during traffic play an essential role (Soane and Ouwerkerk, 1994).

A vast majority of the previous experiments on compaction tended to simulate worst-case scenarios, and some of these studies acknowledged that these conditions do not represent the farmers’ field (Duiker, 2004a; Sidhu and Duiker, 2006). While these scenarios justify the needs and the prevailing state of mechanized farming in relevant study areas (for instance in Europe), the extrapolation of outcomes from these studies becomes limited. Hence, field trafficking employing moderate-to-low axle weight and performed at field moisture contents lower than field capacity on sandy loam soil was investigated to elucidate its impacts on soil physical properties, maize root distribution and consequently on maize growth and yield. Anticipation was that it can help assess the potential threat of compaction at farmers’ field under moderate trafficking conditions and whether planning traffic in better field conditions can mitigate soil compaction.

## 2 Materials and methods

### 2.1 Site description and experimental design

**Figure 1:**
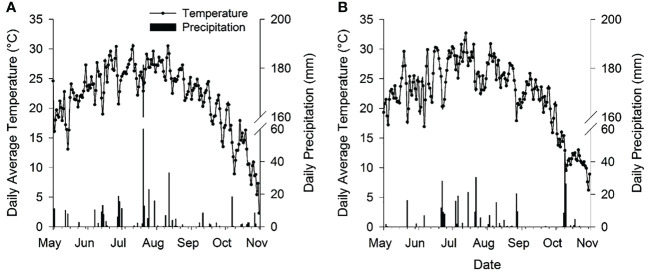
The daily average temperature and daily precipitation during summer maize growing seasons in 2016 **(A)** and 2017 **(B)**.

The experiment was conducted in the summer maize growing seasons of 2016 and 2017 at the Science and Technology Demonstration Garden of the Chinese Academy of Agricultural Sciences located in Langfang (39°07’ N, 116°23’ E), Hebei Province, China. The study site falls in the HHH region of China. The soil texture of the experimental field was sandy loam with 63.7% sand, 21.4% silt, and 16.9% clay. Before starting the experiment, soil bulk density (BD) at 0-15 and 15-30 cm soil layers were 1.37 and 1.46 g cm-3. The soil organic matter, total nitrogen, alkaline-extractable nitrogen, available phosphorus, available potassium, and pH from 0-20 cm soil layer were 6.49 g kg^-1^, 0.63 g kg^-1^, 51 mg kg^-1^, 15.7 mg kg^-1^, 68.3 g kg^-1^, and 7.71, respectively, at the onset of this experiment in 2016. The daily average temperature and precipitation during two growing seasons of maize are shown in **Figure 1**.

The experiment was designed as a randomized complete block under a split-plot arrangement with three replicates. Traffic-induced soil compaction treatments were allotted in main plots, i.e., two-vehicle passes (C2) and six vehicle passes (C6). In contrast, no vehicle pass was considered as control (C0). Two high yielding maize hybrids, i.e., Zhengdan-958 (ZD) and Xianyu-335 (XY), were sown in split plots, each with a net size of 9.6 m × 3.6 m. Maize was hand sown with 40 and 22.5 cm interplant distance in 2016 and 2017, respectively. Low planting density in 2016 was adopted to accommodate the pre-planned (spatial) sampling methodology considering the anticipated crop response; however, recommended planting density was adopted in 2017 after observing the crop response in 2016. Sowing and harvesting were done respectively on 2^nd^ July and 3^rd^ November in 2016 and on 13^th^ June and 13^th^ October in 2017. Fertilizers at rates of 200 kg N ha^-1^ (as urea), 75 kg P_2_O_5_ ha^-1^ (as superphosphate), 100 kg K_2_O ha^-1^ (as potassium sulfate), and 30 kg ZnSO_4_ ha^-1^ were applied in 2016. In 2017, the fertilizers were applied at the rate of 300 kg N ha^-1^, 120 kg P_2_O_5_ ha^-1^, 135 kg K_2_O ha^-1^, and 30 kg ZnSO_4_ ha^-1^. All P, K, and Zn fertilizers (30% in 2016) or (33% in 2017) of N fertilizer were applied at sowing. The rest of N was applied in two splits at V6 (40%) and V12 (27%-30%) stages. The fallow period was observed after the maize harvest. Recommended agronomic practices were followed and no irrigation was used.

### 2.2 Traffic-induced compaction treatment setup

The vehicle employed for compaction purpose was a 60-kW wheel loader, i.e., LG-930 (Shandong Lugong Industry Machinery Co. Ltd.), with a total mass of 5.26 Mg (heavier than normal field tractors used in the region). The load distribution was 60% on the rear axle and 40% on the front axle. All tires were pneumatic tires with a similar size, i.e., 360/70R24 (36 cm tire width, 110.4 cm outer diameter), and were inflated to 120 kPa. The ground contact area of a tire was 0.20 m^2^, calculated as described by Schäfer-Landefeld et al. (2004). The mean ground contact pressure (MGCP) was calculated as (Duiker, 2004a):


[1] MGCP=WL/A

where WL is the load per wheel (kN) and A is the tire contact area (m^2^). The MGCP of the wheel that exerted the maximum stress during a given vehicle pass was 77.5 kPa.

For setting up traffic-induced compaction treatments, adjacent wheel-beside-wheel passes of vehicles were carried out in main plots (74.88 m^2^ excluding buffer zones) in such a way that the whole plot area received the same number of vehicle passes as specified for each treatment. Vehicle passes were zero, two, and six in C0, C2, and C6, respectively. Before the experiment in 2016, the whole experimental area was deeply tilled to a depth of 25 cm using a chisel plough (to remove any previous compaction). Then on June 25, wheel trafficking was performed wheel-beside-wheel across the main plot width. Moisture contents of 0-50 cm soil profile at wheel trafficking was 10.35%-11.75% (w/w) lower than the field capacity. In 2017, no deep tillage was done before wheel trafficking; however, two passes of 10-cm shallow rotary tillage were carried out followed by very light irrigation keeping in view the lesser rain occurrence in preceding months compared to 2016. Afterward, compaction treatments were applied in the same plots and manner on June 8, 2017; and moisture contents of 0-50 cm soil profile ranged 13.75%-14.5% (w/w), which were still lower than the field capacity. One pass of rotary tillage to 10 cm depth was carried out just before sowing to ensure proper germination in both years.

### 2.3 Soil sampling and measurements

The soil BD was determined for each 10-cm soil layer to a depth of 50 cm using the core method and following equation (Blake and Hartge, 1986) at 77 and 88 days after sowing (DAS) in 2016 and 2017, respectively.


. [2] BD = Weight of ovendry soil / Volume of the soil

The total porosity (TP) was calculated as described by Flint and Flint (2002) using following equation:


[3] $$\varphi \;=\;1\;-\;\left(\rho_{b}\;/\;\rho_{p}\right)$$

where *φ* is total porosity; ?*_b_* is the dry bulk density; and ?*_p_*. the particle density of soil. Particle

density of the soil was assumed as 2.65 g cm^-3^.

**Figure 2:**
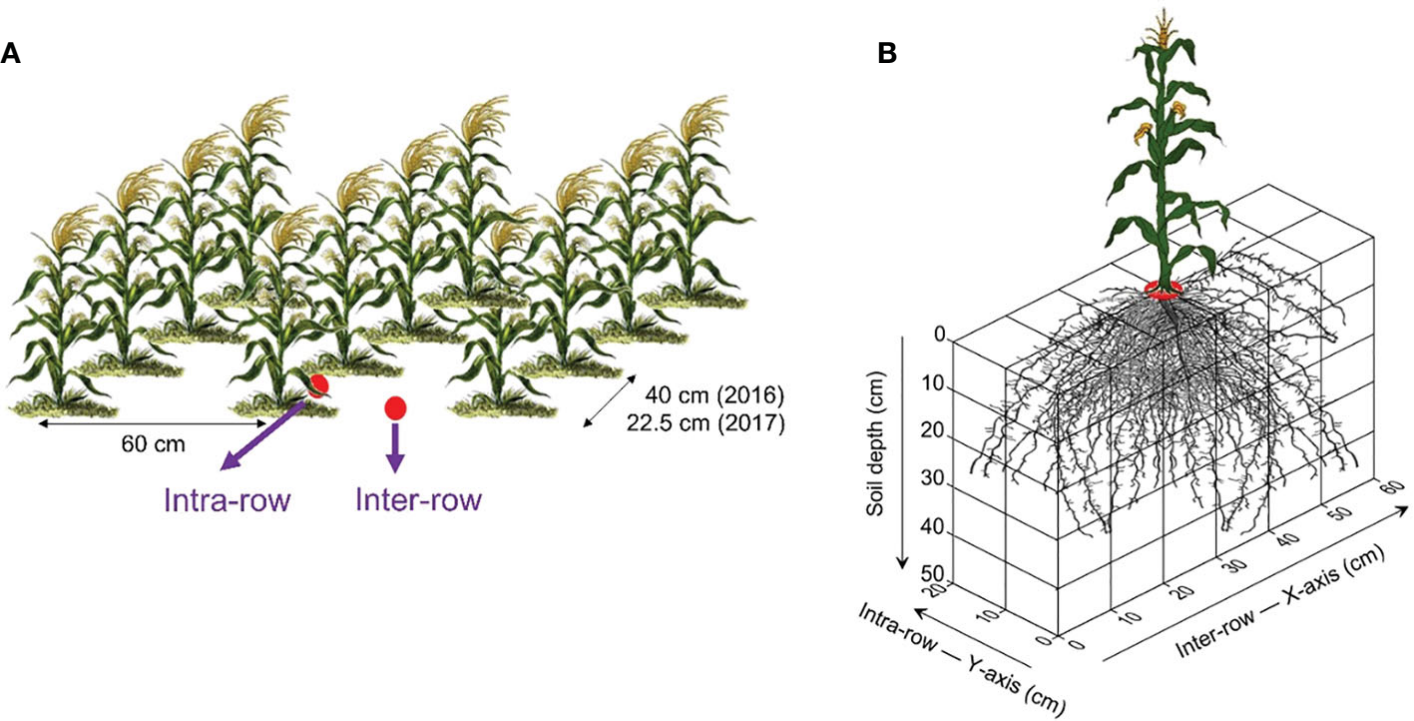
The illustrations of sampling/measurement of soil parameters within experimental plot **(A)** and root sampling method **(B)**.

The penetration resistance (PR) was recorded using hand held digital cone penetrometers (Schäfer-Landefeld et al., 2004; Mu et al., 2016) at 116 and 98 DAS in 2016 and 2017, respectively. Due to unavoidable reasons, two different cone penetrometers were used in the two years. PR values were recorded using a cone-tipped (0.01128 m diameter and 60° angle) penetrometer (Eijkelkamp Agrisearch Equipments, Giesbeek, The Netherlands) up to 80 cm depth in 2016 and a depth of 45 cm at 2.5 cm interval in 2017 using cone-tipped (0.0125 m base diameter and 30° stainless steel cone) digital penetrometer (Field Scout, SC 900 Soil Compaction Meter; Spectrum Technologies, Inc., Plainfield, IL, USA). PR values at depths deeper than 45 cm in 2016 were not considered in analyses of PR values. Six replicate measurements of PR were recorded for each inter-and intra-row position within a plot (**Figure 2A**). The mass-based gravimetric soil moisture contents (g g^-1^) were also determined for each 10-cm layer to a depth of 50 cm. As the moisture contents at the time of PR measurements were uniform, the PR data were not adjusted for soil moisture content (Gregorich et al., 2011).

For statistical analysis, the PR values were averaged across 10-cm layers. Furthermore, PR measurements were also analyzed for the following soil resistance indices (Tekeste et al., 2008; Sivarajan et al., 2018): average PR up to 45 cm (APR_FP_); maximum PR value (PR_MAX_); depth to maximum PR value (DMPR); depth to the top of the hardpan layer (DTHP); corresponding PR value at the top of the hardpan layer (PR_THP_); and average PR in hardpan layer up to 45 cm (APR_HP_). Depth from the soil surface to the depth of maximum PR in 0-45 cm soil profile was considered as depth to full PR. The start of the hardpan layer was assumed where PR value increased rapidly with the change in slope. Average PR from the top of the hardpan layer up to 45 cm was treated as average PR in the hardpan. The relation of BD and PR to crop variables in 0-30 cm soil layers (topsoil) were calculated by taking weighted means of the corresponding values of 0-10, 10-20, and 20-30 cm layers using weights of respectively 2, 2, and 1 (Gregorich et al., 2011).

At 68 and 70 DAS in 2016 and 2017, water-stable aggregate size distribution (WASD) was determined for inter and intra row positions (**Figure 2A**) as described by Cambardella and Elliott (1993) and Nimmo and Perkins (2002). Briefly, a 50 g air dried subsample was wet sieved through three nested sieves (in order from the top) of 2, 0.25, and 0.053 mm. The soil was evenly spread on top of the nest of sieves and suspended in the distilled water for five minutes. Afterward, sieves were moved 3.8 cm vertically through the water at 30 cycles per minute for 10 minutes. The soil that remained on each sieve was collected and dried. Resultantly, four aggregate size fractions were obtained which were (i) >2 mm (large macroaggregates), (ii) 0.25–2 mm (small macroaggregates), (iii) 0.053–0.25 mm (microaggregates), and (iv) <0.053 mm (silt and clay-size particles). Sand corrections were made using sodium hexametaphosphate as dispersing agent (Nimmo and Perkins, 2002). For the mathematical representation of WASD, the mean weigh diameter (MWD) was calculated as (Kemper and Rosenau, 1986):


[4] $$MWD=\;\sum_{i=0}^{n}w_{i}{\bar{X}}_{i}$$

where ${\bar{X}}_{i}$ is the arithmetic mean diameter of each size fraction (mm); *w_i_* is the proportion of the total water-stable aggregates in the corresponding size fraction, and *n* is the number of all size fractions.

### 2.4 Plant sampling and measurements

Three plants from each plot were harvested at 35, 63, and 112 DAS in 2016 and at 33, 45, 65, and 96 DAS in 2017 to determine green leaf area index (LAI) and aboveground biomass (AGB). Length (L) and maximum width (W) of each green leaf was used to calculate leaf area (LA) (Montgomery, 1911):


[5] LA=L ×W ×0.75

LAI was calculated as:


[6] LAI=(LA ×PD)/10000

where LA is the leaf area (m^2^ plant^-1^) and PD is the planting density (plants ha^-1^). Afterward, the plants were separated into leaves, sheaths, stem, cob husk, and ear and oven-dried to a constant weight at 70°C for AGB estimation.

Furthermore, the final AGB was determined at physiological maturity by harvesting six representative plants from the central harvest area of each plot. Ears were carefully removed and husks were included in stover. Grain yield and yield components were determined by manually harvesting ears from five 3-m long rows of harvest area. Grain moisture contents were measured and grain yield was reported at 15.5% moisture content. The number of kernels per ear were recorded from six ears, and the weight of 100 kernels was determined by oven-drying at 70°C.

In 2017, root sampling was done at 78 DAS as per the three-dimensional (3D) spatially distributed soil monolith excavation method (**Figure 2B**). From each 10 cm soil layer, 12 soil monoliths, each of 10×10×10 cm^3^ volume were excavated from an area of 60 cm × 20 cm with a plant positioned at the center, and sampling was done to a depth of 50 cm. Roots were washed and collected from each monolith after the soil passed through a 0.5 mm sieve using a hose and nozzle attachment. Roots were then scanned using a scanner (Epson V700, Germany) and the images obtained were analyzed for root length using WinRhizoPro 5.0 software (Regent Instruments Inc., Quebec, Canada). Afterward, root samples were oven dried at 70°C. Root mass density (RMD) (Mu et al., 2016) and root length density (RLD) (Liu et al., 2017) were calculated as:


[7] RMD=root dry weight / soil volume

[8] RLD=root length / soil volume

Root distribution ratios, i.e., root dry weight ratio (RDR), and root length ratio (RLR) were calculated by dividing root dry weight or length in each soil layer by total root dry weight or length, respectively (Osaki et al., 1995). Furthermore, visual observations were also recorded for visible root growth and penetrability by digging trenches and excavating whole root systems.

### 2.5 Statistical analysis

One-way analysis of variance (ANOVA) with Fisher’s least significance (LSD) tests at a significance level of P < 0.05 was used to evaluate all the data. The uniformity and normality of the variances were determined using the Levene and Shapiro-Wilk tests prior analysis. Data were subjected to the analysis of variance (ANOVA) using the Proc GLM procedure of SAS, Version 9.2 (SAS Institute Inc. Cary, NC, USA). Years were analyzed separately because of the difference in compaction treatment intensity (i.e., 1 year vs. 2 years of compaction) and due to changes in planting density. Compaction levels, cultivars, and their interaction were taken as fixed effects in the model, whereas experimental blocks and their interaction were considered random effects. Furthermore, appropriate error terms were used for hypothesis testing for main plots, sub-plots, and their interaction effects. Appropriate comparisons were made for interaction effects wherever significant. Spatial distribution of root length was visualized using Surfer16.0 software (Golden Software LLC, CO, USA). Correlation and regression analyses were performed using the R program and SigmaPlot, Version 12.5 (Systat Software Inc., Chicago, IL, USA).

## 3 Results

### 3.1 Soil variables

#### 3.1.1 Bulk density

The effect of traffic-induced compaction on soil BD is shown in **Table 1**. With the increase in compaction level, BD significantly increased (i.e., C6 > C2 > C0) at 10-30 cm and 0-30 cm soil layers in 2016 and 2017, respectively. The most significant effect on BD was observed in the 10–20 cm soil layer, where compaction-caused increases, in comparison with control, were 8.96–12.69% in 2016 and 14.18–16.42% in 2017, respectively.

**Table 1:** The mean values of soil bulk density at various soil depths for three compaction levels in 2016 and 2017.

Year	Treatment	0-10 cm	10-20 cm	20-30 cm	30-40 cm	40-50 cm
2016	C0	1.35 a	1.34 b	1.46 b	1.51 a	1.43 a
	C2	1.36 a	1.46 a	1.51 ab	1.51 a	1.46 a
	C6	1.37 a	1.51 a	1.54 a	1.53 a	1.46 a
	*p-value C*	*0.4723*	0.0032**	0.0406*	*0.6311*	*0.4536*
2017	C0	1.32 b	1.34 c	1.51 b	1.49 a	1.41 a
	C2	1.34 b	1.53 b	1.52 b	1.50 a	1.46 a
	C6	1.38 a	1.56 a	1.56 a	1.52 a	1.46 a
	*p-value C*	0.012*	<0.0001***	0.0173*	*0.7335*	*0.062*

C0, control without trafficking; C2, two traffic passes; C6, six traffic passes; C, compaction factor.

Within a treatment factor, means followed by the same letter do not differ significantly according to analysis of variance (ANOVA) and LSD post hoc test at p < 0.05. *, **, and *** highlight p-values lower than 0.05, 0.01, and 0.001, respectively.

#### 3.1.2 Penetration resistance

**Figure 3:**
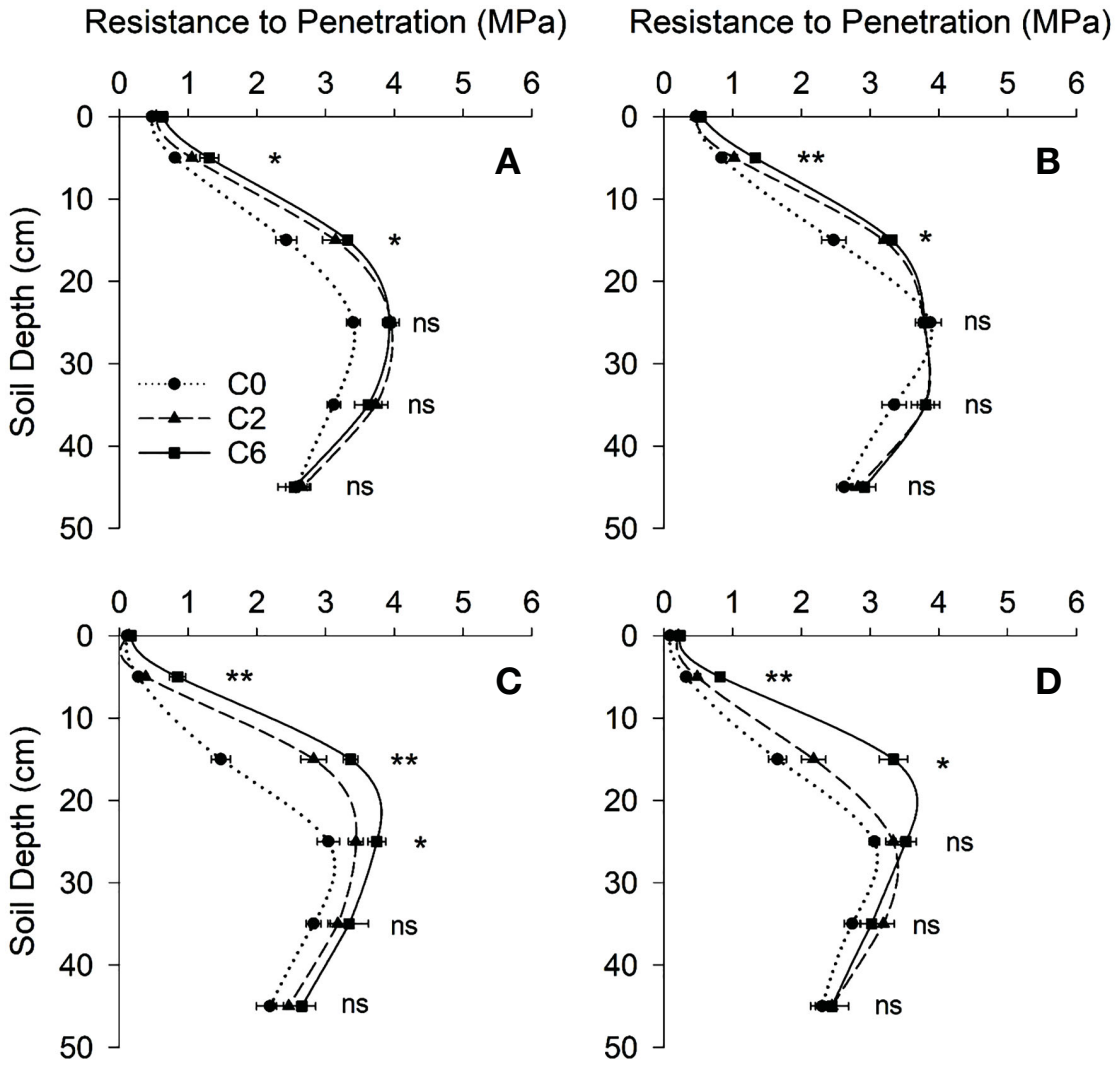
Soil resistance to penetration with depth under different compaction treatments in 2016 **(A, B)** and 2017 **(C, D)** at intra- and inter-row positions, respectively. Symbols represent mean values of different compaction treatments at each depth; error bars represent standard error of means. Treatments: C0, control; C2, two traffic passes; C6, six traffic passes. *, and ** indicate significant differences within the same soil layer at respectively p < 0.05, and p < 0.01 as per ANOVA results; ns indicates no significant difference.

**Figure 4:**
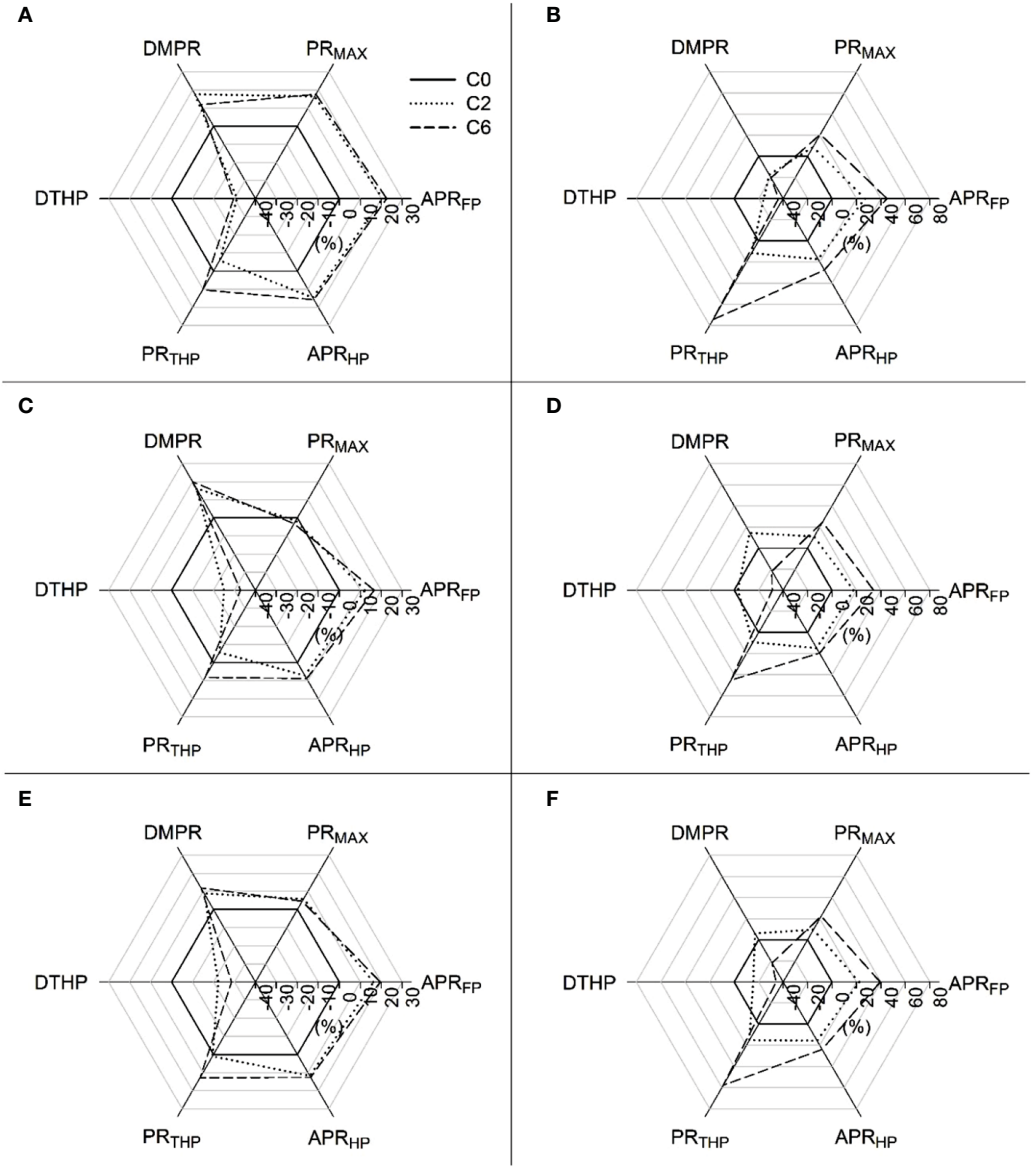
Comparison of penetration resistance indices under different compaction treatments with control considering their relative values at intra- **(A, B)** and inter-row **(C, D)** positions and average **(E, F)** in 2016 **(A, C, E)** and 2017 **(B, D, F)**, respectively. Treatments: C0, control; C2, two traffic passes; C6, six traffic passes. Penetration resistance (PR) indices: APR_FP_, average PR up to 45 cm; PR_MAX_, maximum PR value; DMPR, depth to maximum PR value; DTHP, depth to the top of the hardpan layer; PR_THP_, corresponding PR value at the top of the hardpan layer; APR_HP_, average PR in hardpan layer up to 45 cm.

Averaged PR values for each 10-cm soil layer, measured at intra-row positions (**Figure 3**) show that PR values in 2016 were significantly lower for control only in the top two 10-cm layers, whereas significantly lower PR values in control were found in 0-10, 10-20, and 20-30 cm layers in 2017. The most prominent difference was seen in 10-20 cm layer where C2 and C6 recorded respectively 91.40% and 127.76% higher PR in 2017. Compaction significantly increased APR_FP_ and APR_HP_ but decreased the DTHP in both years, and all studied PR indices except DMPR were influenced by compaction in 2017 (**Figure 4**). C6 treatment recorded maximum APR_FP_ and APR_HP_. Compaction not only moved the hardpan to shallower depths (up to 34% decrease in DTHP over control) but also increased soil strength at the top of the hardpan (up to 58% increase in PR_THP_ over control) and within the hardpan (up to 24% increase in APR_HP_ over control).

#### 3.1.3 Soil moisture and aggregate size distribution

**Figure 5:**
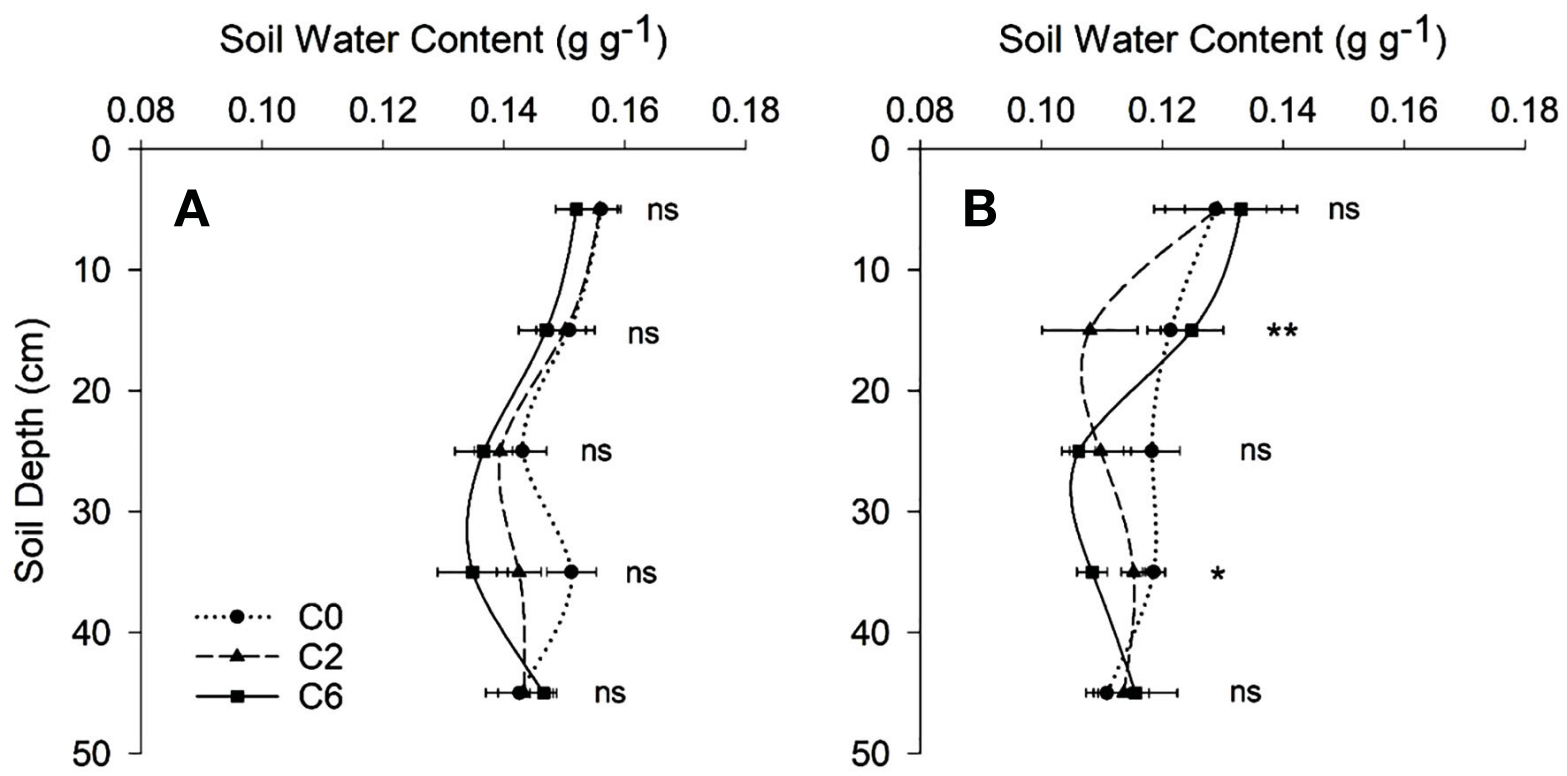
Soil moisture contents with depth under different compaction treatments in 2016 **(A)** and 2017 **(B)**. Symbols represent mean values of different compaction treatments at each depth; error bars represent standard error of means. Treatments: C0, control; C2, two traffic passes; C6, six traffic passes. *, and ** indicate significant differences within the same soil layer at respectively p < 0.05, and p < 0.01 as per ANOVA results; ns indicates no significant difference.

**Figure 6:**
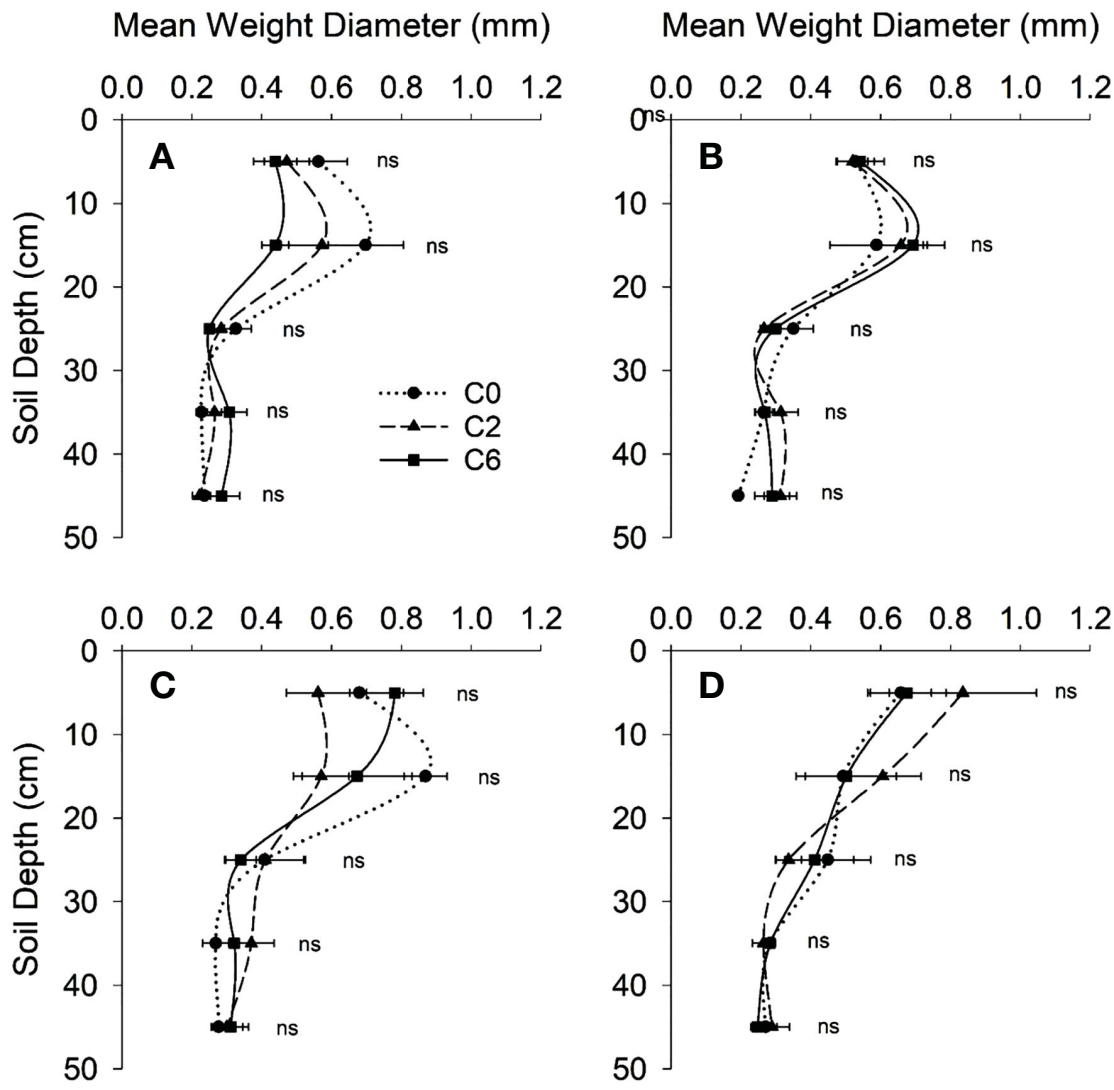
Mean weight diameter of water-sTABLE aggregates with depth under different compaction treatments in 2016 **(A, B)** and 2017 **(C, D)** at intra- **(A, C)** and inter- **(B, D)** row positions, respectively. Symbols represent mean values of different compaction treatments at each depth; error bars represent standard error of means. Treatments: C0, control; C2, two traffic passes; C6, six traffic passes. ns indicates no significant difference as per ANOVA results.

The soil moisture contents did not vary significantly among treatments at any soil depth except at 10-20 and 30-40 cm layers in 2017, where the maximum difference was just 0.017 g g^-1^ (**Figure 5**). In both years, MWD was not significantly affected by treatments; however, average values of MWD appeared to be higher in control for an intra-row position in 0-10 cm (only in 2016) and 10-20 cm (in both years) soil layers than that in C2 or C6 (**Figure 6**).

### 3.2 Plant Variables

Like soil variables, no significant interaction effect on plant variables was found in general.

#### 3.2.1 Leaf area index and aboveground dry matter

**Figure 7:**
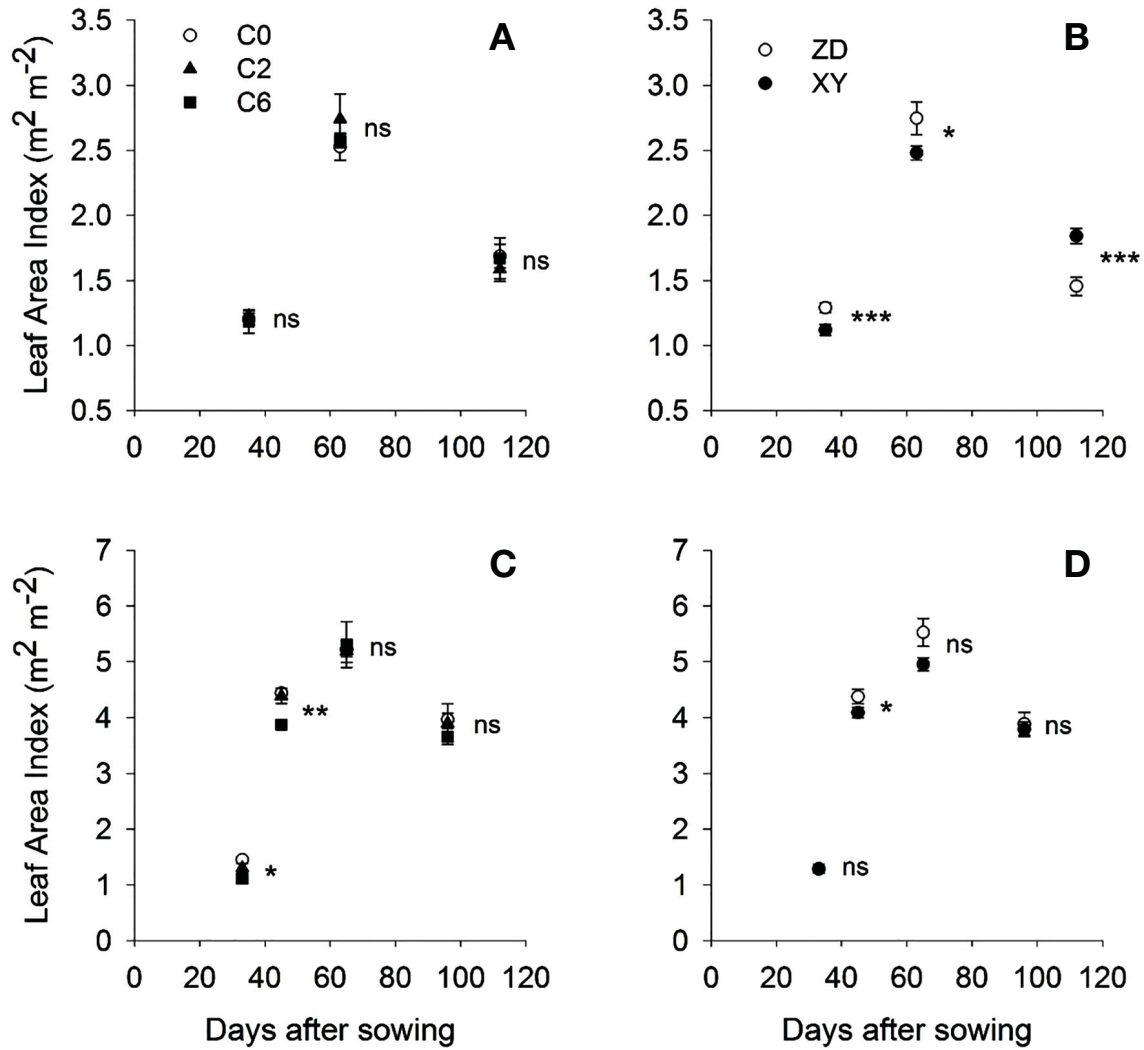
Leaf area index for different compaction treatments **(A, C)** and cultivars **(B, D)** at various growth stages in 2016 **(A, B)** and 2017 **(C, D)**, respectively. Symbols represent mean values of different treatments; error bars represent standard error of means. Treatments: C0, control; C2, two traffic passes; C6, six traffic passes; ZD, Zhengdan-958; XY, Xianyu-335. *, **, and *** indicate significant differences within the same growth stage at respectively p < 0.05, p < 0.01, and p < 0.001 as per ANOVA results; ns indicates no significant difference.

Compaction treatments showed no significant effect on LAI in 2016; however, significant effects were observed in 2017, mainly at early crop growth stages (**Figure 7**). LAI in C6 was significantly lower than those in statistically similar C0 and C2 by respectively 23.45% and 14.62% at 33 DAS and 12.84% and 11.84% at 45 DAS for 2017. Meanwhile, ZD tended to have higher LAI than XY at most growth phases in two years.

Significantly lowest aboveground dry matter (ADM) was found in C6 than in C0 and C2. Compared with control in 2017, ADM in C2 and C6 were 11.69% and 19.03% lower, respectively. For maize variety, XY tended to have higher ADM than ZD at most of the growth phases in 2017.

#### 3.2.2 Root growth and distribution

**Figure 8:**
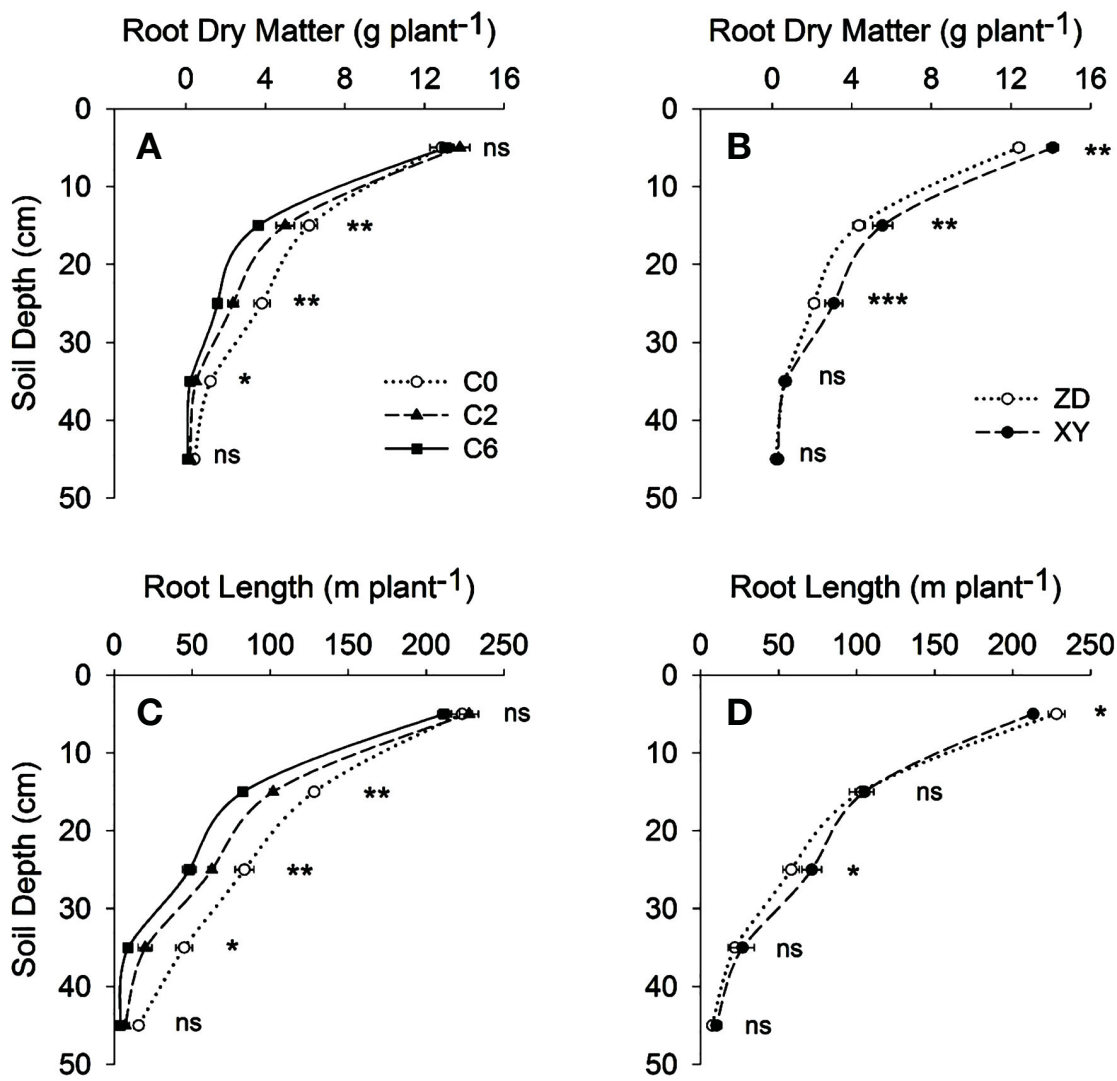
Root dry matter **(A, B)** and root length **(C, D)** with soil depth under different compaction treatments and cultivars, respectively, in 2017. Symbols represent mean values of different treatments at each depth; error bars represent standard error of means. Treatments: C0, control; C2, two traffic passes; C6, six traffic passes; ZD, Zhengdan-958; XY, Xianyu-335. *, **, and *** indicate significant differences within the same soil depth at respectively p < 0.05, p < 0.01, and p < 0.001 as per ANOVA results; ns indicates no significant difference.

**Figure 9:**
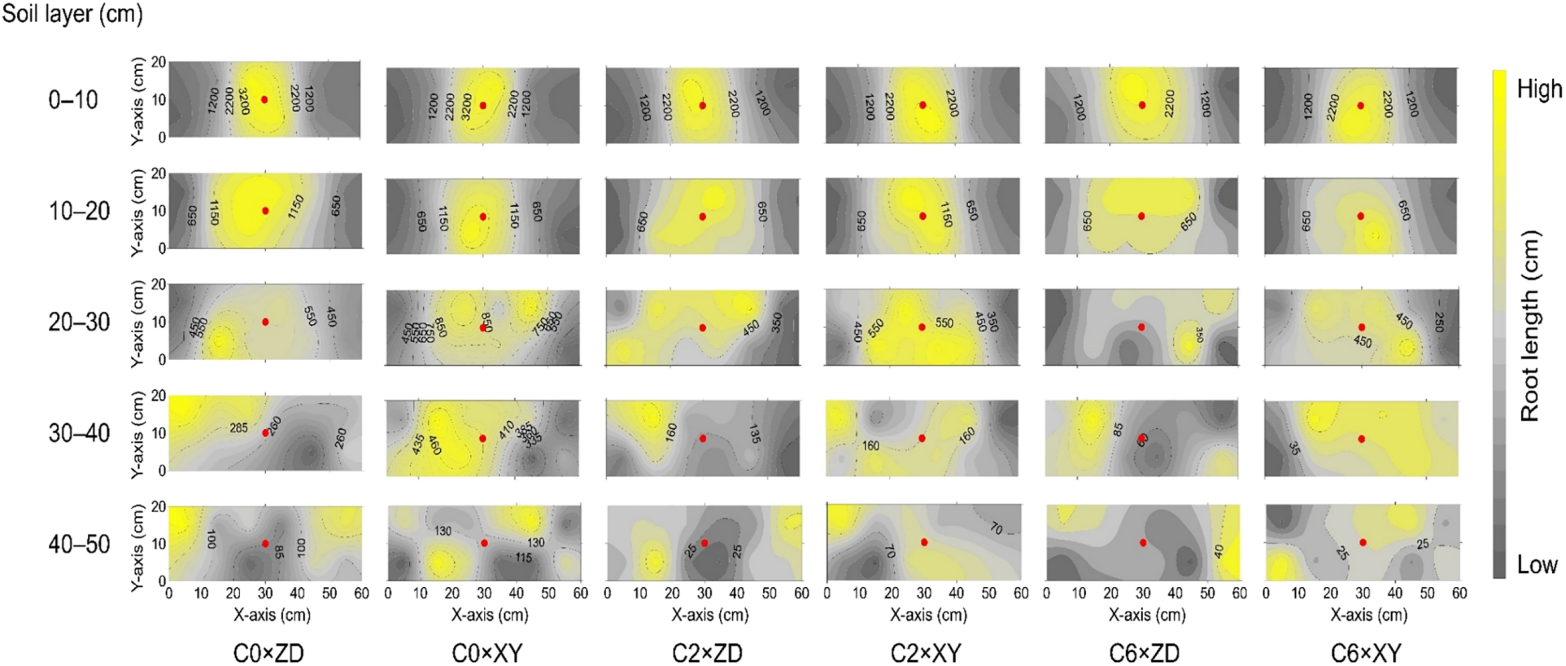
Three-dimensional distribution of root length under different compaction treatments and cultivars in 2017. Root length values are mean of three replicates. X-axis and Y-axis represent inter- and intra-row directions, whereas the red dot represents the plant position. Treatments: C0, control; C2, two traffic passes; C6, six traffic passes; ZD, Zhengdan-958; XY, Xianyu-335.

Root investigation in 2017 revealed that compaction substantially impaired root growth and altered root distribution within the soil. Compared with the control (C0), the per plant root dry matter and length were respectively 24.09% and 28.33% less in C6 (**Figure 8**). 3D spatial distribution of root length also indicated that compaction not only reduced root growth but also promoted horizontal root proliferation (**Figure 9**).

**Figure 10:**
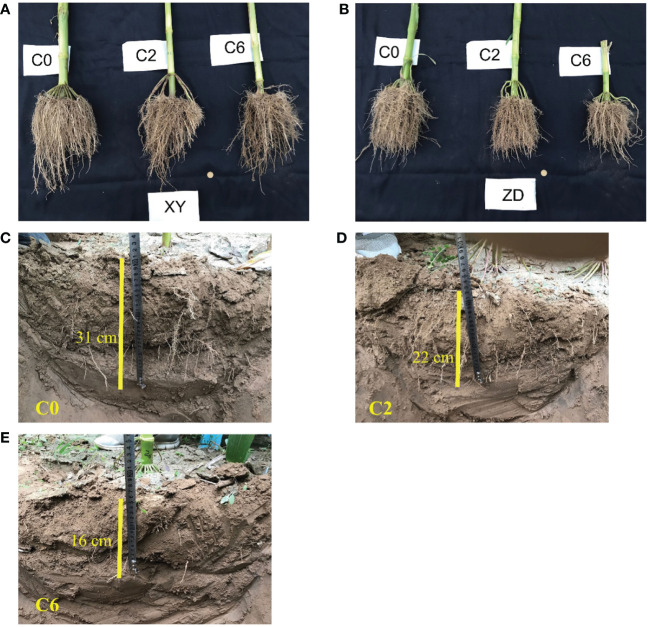
Excavated whole roots **(A, B)** and depth to visible roots in trench **(C–E)** under different compaction treatments. Root growth under different compaction levels (C0, control; C2, two traffic passes; C6, six traffic passes) is shown in **(A, B)** for the two maize cultivars XY (Xianyu-335) and ZD (Zhengdan-958), respectively. Depths to visible roots after digging trench in middle of two maize rows for C0, C2, and C6 are shown in **(C–E)**, respectively.

Regarding vertical root distribution, the reductions in RMD and RLD due to compaction increased with increasing soil depth (**Table 2**). In the 20-30 cm soil layer, C0×XY had significantly higher RMD, followed by statistically similar C0×ZD and C2×XY. Compared with C0, RLD was 80.46% lower in the 30-40 cm layer under C6. Compared with control, C2 and C6 had higher RDR and RLR in the 0-10 cm soil layer; however, RDR and RLR decreased as compaction levels increased at all other soil depths (**Table 3**). The RLR in 10-20 cm soil layer was highest in C0×ZD followed by C2×XY; however, compaction did not significantly reduced RLR for XY in this layer. In 30-40 cm soil layer, RLR was highest in C0×XY and significant reductions were observed under compaction. Visual observations regarding root growth and penetrability revealed that generally more roots were visible at deeper soil depths in C0 as compared to C2 and C6 suggesting reduced root growth and penetrability under compacted treatments (**Figure 10**). Regarding cultivars, XY appeared to have deeper roots than ZD under all compaction levels.

**Table 2:** Aboveground dry matter accumulation for three compaction levels and two maize cultivars at different plant growth stages in 2016 and 2017.

Treatment	2016				2017			
	35 DAS	63 DAS	112 DAS	33 DAS	45 DAS	65 DAS	78 DAS	96 DAS
C0	30.95 a	180.55 b	345.76 a	14.55 a	65.50 a	179.55 a	237.93 a	318.91 a
C2	31.93 a	195.55 a	352.42 a	12.27 ab	64.20 a	166.59 a	226.77 a	305.98 ab
C6	30.02 a	199.66 a	342.38 a	10.43 b	51.41 b	160.43 b	185.41 b	265.90 b
*p-value C*	*0.7403*	0.0356*	*0.7818*	0.0342*	0.0044**	*0.2467*	0.0121*	0.0471*
ZD	31.61 a	197.88 a	346.90 a	12.64 a	60.46 a	151.45 b	200.03 b	280.78 b
XY	30.32 a	185.97 a	346.81 a	12.20 a	60.28 a	186.26 a	233.38 a	313.08 a
*p-value V*	*0.2232*	*0.135*	*0.9943*	*0.6401*	*0.9402*	0.0135*	0.0011**	0.0018**
*p-value C × V*	0.0406*	*0.3373*	*0.6769*	*0.223*	*0.504*	*0.7974*	*0.2432*	*0.1006*

C0, control without trafficking; C2, two traffic passes; C6, six traffic passes; ZD, Zhengdan-958; XY, Xianyu-335; C, compaction factor; V, Cultivar factor; and DAS, days after sowing.

Within a treatment factor, means followed by the same letter do not differ significantly according to analysis of variance (ANOVA) and LSD post hoc test at p < 0.05. * and ** highlight p-values lower than 0.05 and 0.01, respectively.

**Table 3:** Mean values of root dry weight and length ratios in different soil layers for three compaction levels and two maize cultivars at grain filling stage in 2017.

Treatments	Soil depth					
	0-10 cm	10-20 cm	20-30 cm	30-40 cm	40-50 cm	0-50 cm
Root dry weight ratio (%)						
C0	52.60 c	25.27 a	15.39 a	5.01 a	1.73 a	93.26 b
C2	63.41 b	22.69 a	10.77 b	2.23 b	0.89 a	96.88 ab
C6	70.27 a	19.51 b	8.49 c	1.18 b	0.55 a	98.27 a
*p-value C*	0.0019**	0.0142*	0.0012**	0.0314*	*0.1519*	0.0458*
ZD	63.56 a	21.97 a	10.50 b	3.02 a	0.96 a	96.02 a
XY	60.63 b	23.01 a	12.60 a	2.60 a	1.15 a	96.25 a
*p-value V*	0.0132*	*0.2306*	0.0016**	*0.3197*	*0.5941*	*0.7321*
*p-value C × V*	*0.3712*	*0.8416*	*0.3056*	*0.84*	*0.5019*	*0.7214*
Root length ratio (%)						
C0	45.10 c	25.94 a	16.88 a	8.97 a	3.10 a	87.92 b
C2	54.38 b	24.36 ab	14.99 a	4.62 b	1.65 a	93.73 a
C6	59.61 a	23.25 b	13.55 a	2.45 b	1.13 a	96.42 a
*p-value C*	0.0002***	0.0252*	*0.1657*	0.023*	*0.1606*	0.0308*
ZD	55.15 a	24.42 a	13.75 b	4.99 a	1.71 a	93.31 a
XY	50.92 b	24.62 a	16.53 a	5.71 a	2.22 a	92.07 a
*p-value V*	0.0056**	*0.6175*	0.0302*	*0.1860*	*0.3741*	*0.2366*
*p-value C × V*	*0.7677*	0.0094**	*0.4713*	0.0330*	*0.5043*	*0.1667*

C0, control without trafficking; C2, two traffic passes; C6, six traffic passes; ZD, Zhengdan-958; XY, Xianyu-335; C, compaction factor; and V, Cultivar factor.

Within a treatment factor, means followed by the same letter do not differ significantly according to analysis of variance (ANOVA) and LSD post hoc test at p < 0.05. *, **, and *** highlight p-values lower than 0.05, 0.01, and 0.001, respectively.

#### 3.2.3 Grain yield and yield components

Kernels per ear were not affected by compaction in 2016; however, kernels per ear were 18.67% higher under C0 than those under C6 in 2017 (**Table 4**). ZD had higher kernels per ear than XY in both years. Compaction did not affect 100-kernels weight in both years; however, XY recorded a 10.14% higher 100-kernels weight than ZD in 2017. Though not significant, HI was highest in C2.

**Table 4:** Ear number, kernel number (per ear and per unit area), 100-Grain weight (oven dry), grain yield (15.5% moisture), and harvest index for three compaction levels and two maize cultivars in 2016 and 2017.

Treatments	Ear number	Kernel number	Kernels number	100-Grain weight	Grain yield	Harvest index
	(Ears ha^-1^)	(Kernels ear^-1^)	(Kernels m^-2^)	(g)	(Mg ha^-1^)	(%)
2016						
C0	42708.33 a	482.47 a	2060.77 b	30.01 a	7.31 b	49.07 a
C2	44965.28 a	498.03 a	2229.77 a	29.52 a	7.78 a	49.42 a
C6	44444.44 a	475.77 a	2103.31 b	29.62 a	7.36 b	46.80 a
*p-value C*	*0.2298*	*0.2491*	0.0070**	*0.5117*	0.0040**	*0.1355*
ZD	46412.04 a	466.73 b	2161.30 a	29.37 a	7.50 a	50.05 a
XY	41666.67 b	504.12 a	2101.26 a	30.06 a	7.46 a	46.82 b
*p-value V*	0.0004***	0.0088**	*0.0512*	*0.0662*	*0.7145*	0.0075**
*p-value C × V*	*0.4602*	*0.067*	0.0351*	*0.4027*	*0.0609*	*0.8709*
2017						
C0	73884.14 a	534.34 a	3948.35 a	31.65 a	14.77 a	49.02 a
C2	74074.07 a	496.61 b	3678.62 b	31.46 a	13.65 b	51.03 a
C6	73504.27 a	449.62 c	3304.86 c	31.99 a	12.48 c	50.96 a
*p-value C*	*0.1736*	0.0059**	0.0065**	*0.6511*	0.0117*	*0.1693*
ZD	73820.83 a	499.09 a	3685.39 a	30.17 b	13.13 b	52.10 a
XY	73820.83 a	487.96 a	3602.49 a	33.23 a	14.14 a	48.58 b
*p-value V*	*1.0000*	*0.3736*	*0.3515*	0.0031**	0.0073**	0.0034**
*p-value C × V*	*0.2963*	*0.7104*	*0.7852*	*0.3490*	*0.0638*	*0.5893*

C0, control without trafficking; C2, two traffic passes; C6, six traffic passes; ZD, Zhengdan-958; XY, Xianyu-335; C, compaction factor; and V, Cultivar factor.

Within a treatment factor, means followed by the same letter do not differ significantly according to analysis of variance (ANOVA) and LSD post hoc test at p < 0.05. *, **, and *** highlight p-values lower than 0.05, 0.01, and 0.001, respectively.

There were significant differences among compaction treatments for grain yield in both years; however, the trend and magnitude of differences were not the same in the two years. In 2016, the lowest recorded yield (in C0×XY) was 9.23% lower than the highest recorded yield (in C2×ZD). Averaged across cultivars, grain yield in C2 was 6.43% and 5.70% higher than that in C0 and C6, respectively. In 2017, however, yield reductions in C2 and C6 treatments were 7.58% and 15.50%, respectively, compared with control. C0×XY recorded the highest grain yield (15.82 Mg ha^-1^), whereas C6×ZD recorded the lowest yield in 2017.

## 4 Discussion

### 4.1 Locale of soil compaction

In the field machinery operation, subsoil compaction is controlled by axle load, whereas topsoil compaction is governed by ground contact pressure (Botta et al., 1999 and Duiker, 2004b; Botta et al., 2008). A recent study on sandy loam soils concluded that approximately 3 Mg wheel load (6 Mg axle load) is the upper threshold limit to avoid subsoil compaction at highly inflated tire pressure (Schjønning et al., 2016b). Thus, in this study, the low axle load (3.16 Mg) used did not cause any subsoil compaction, but it induced compaction (as indicated by BD and PR) in the topsoil (0-30 cm) profile. Sweeney et al. (2006) also did not find the effect of wheel traffic below 20 cm soil depth. Meanwhile, the compaction in the surface soil layer (0-10 cm) was somewhat mended by a single pass of rotary tillage performed just before sowing, only leaving effective compaction in 10-30 cm soil layers. Therefore, soil tillage has the potential to mitigate soil compaction (Shen et al., 2016), which is further assisted by natural freeze-thaw cycles (Sivarajan et al., 2018) and other factors like drying-wetting of soil and functions of soil biota (Schjønning et al., 2016a).

### 4.2 Number of traffic passes and carryover effect

Initial traffic passes caused significant damage as the increase in BD after two vehicle passes (C2) was 71%-86% of the maximum increase observed under six vehicle passes (C6) in the 10-20 cm layer (**Table 1**). Generally, much of the compaction occurs after the first pass (Duiker, 2004a). However, a significant difference between C2 and C6 in 0-30 cm soil profile in 2017 pointed toward the harmful effects of repeated traffic (Botta et al., 1999). The impact of multiple passes was also evident from the fact that C6 differed significantly from C0 even in the 0-10 cm layer, where tillage alleviated much of the traffic-induced compaction. More pronounced effects of field trafficking in 2017 can be attributed partly to the residual effects of field traffic in the previous year, as effects of compaction can be persistent even in upper 30 cm soil layers (Berisso et al., 2012). Gregorich et al. (2011) also pointed out the carryover effect of traffic on PR. Moreover, relatively higher soil moisture content at the time of field trafficking in 2017 and the absence of deep plowing before traffic application, in contrast to 2016, might have contributed to more pronounced effects in 2017.

### 4.3 Effect on penetration resistance and soil hardpan

PR is well-known to evaluate mechanical impedance to root development (Keller et al., 2015; de Lima et al., 2016). In the current study, the PR values surpassed 2 MPa value at soil depths below 20 cm, even under C0. The PR value of 2 MPa is often considered a critical value that limits root growth (da Silva et al., 1994); however, many studies have disputed this by considering different critical values (Lipiec and Hatano, 2003; Botta et al., 2004; Chen et al., 2014). In this study, relatively lower moisture contents explain the range of PR measurements (**Figure 5**). Similar to those in this study, low moisture contents led to higher PR values than often reported critical values (Botta et al., 2016). Due to factors like compression beneath and around the penetrometer tip, which can be more important under low moisture contents, and the dependency of the force of penetration on soil density, the trend in the angle of internal friction is that soil resistance increases as the moisture level decreases (Botta et al., 2016). In addition to the substantial influence of BD, moisture contents, and soil texture, the interaction between soil and cone penetrometer can also affect PR values (Lunne et al., 1997), as suggested by the slight variation in two years’ values involving two different penetrometers. Hence, the PR values, including the often-quoted critical value, should not be read exclusively while considering the impact on plant growth as damage to plant roots can be less than suggested by these values. However, looking more profound than mere PR values, the PR profiles can help assess the overall growing conditions surrounding the root. Various PR indices, such as used in this study, are often used to characterize hardpan and evaluate the effects of field traffic on soil PR (Tekeste et al., 2008; Sivarajan et al., 2018). The slight variations at inter and intra row positions for PR indices suggested the effects of roots and root activity. The PR indices indicated that field trafficking resulted in shallower and stronger hardpan entailing difficulty in root penetration. Nevertheless, PR measurements were complemented by investigating root parameters and crop variables due to the reasons commented above.

### 4.4 Severity of compaction under dry field conditions

No significant effect of trafficking on MWD suggests that traffic-induced compaction was not so severe to deform soil aggregates substantially (**Figure 6**). A plausible reason is low soil moisture level at the time of trafficking because the extent of aggregate deformation is strongly associated with wetter soil conditions (Wolkowski, 1990; Batey, 2009; Shah et al., 2017). Moreover, the soil type was sandy loam, and wet clayey soils are usually more prone to severe compaction effects than dry sandy soils.

### 4.5 Differences in crop response to field traffic in first and second year

Though the crop response to field traffic in 2016 seems to contradict general anticipation and what was observed in 2017, it is not inexplicable or unexampled. Some studies also found no significant effect of traffic-induced compaction on maize yield (Gelder et al., 2007; Sivarajan et al., 2018). In current study, the soil was deeply ploughed to approximately 25 cm before applying field traffic in 2016. This was exactly the case in series of experiments analyzed by Arvidsson and Håkansson (2014). They found some evidence about yield increment under moderate compaction compared with non-trafficked and previously loosened soil. Arguably, enhanced nutrient and water uptake due to improved root-soil contact and increased unsaturated hydraulic conductivity can explain the positive effects under moderate compaction (Lipiec and Hatano, 2003; Blanco-Canqui and Lal, 2007). The extents of traffic-induced alterations in soil properties were lesser in 2016, and most of the crop growth variables and yield in 2016 did not show a significant correlation with BD or PR. Thus, traffic-induced moderate re-compaction after deep tillage in 2016 might have reduced nutrient leaching considering the sandy loam type of soil. Moreover, the planting density in 2016 was relatively lower (lower than recommended) and reduced inter-plant competition might have been a reason for no adverse effects of field trafficking.

The reasons mentioned above were further strengthened by observing the harmful effects of compaction in 2017 when field trafficking was not preceded by deep tillage and planting density was higher. In addition, a higher magnitude of increases in BD and PR indicated carry over effect of field traffic (Gregorich et al., 2011; Gregorich et al., 2014). Thus, we infer that the carry over effect of compaction in the upper 30 cm soil profile contributed to effectuate yield penalties in the second year of annual trafficking. This is in accordance with a previous study in which it was reported that it took until the third year of annually repeated field trafficking to cause significant yield reductions (Sweeney et al., 2006). In the current study, the maximum yield reduction of 15.5% under C6 was comparable to earlier reports such as Tolon-Becerra et al. (2011) and Sidhu and Duiker (2006).

Nevertheless, much higher yield penalties in maize have also been reported. For instance, maize yield reductions of up to 43% were due to subsoil compaction when an 11 Mg axle load was employed by Voorhees (2000). However, this axle load is almost 3.5 times the axle load used in the current study. An increase in yield penalty with an increased number of the vehicle passes was confirmed as yield penalty doubled in C6 compared with C2 (Zhang et al., 2006).

### 4.6 Compaction affects crop growth more at early stages

Though the negative effects of compaction on LAI diminished as the crop season progressed, the early setbacks caused by compaction led to lower dry matter accumulation per plant. Up to 19% compaction-induced reductions in dry matter yield in the current experiment were consistent with previous reports (Chen and Weil, 2011; Gregorich et al., 2011). Soil compaction affects various plant physiologic functions especially at early growth stages (Mirleau-Thebaud et al., 2017; Martina et al., 2018). Tubeileh et al. (2003) found that compaction decreased the carbon assimilation rate in maize at early growth stages, resulting in reduced leaf emergence rate, leaf area, plant height, and shoot biomass, and the effects persisted as the crop growth progressed. Though plants tended to overcome adverse effects inflicted at early stages, the effects still translated to the grain yield.

### 4.7 Compaction-induced alterations in root growth and distribution effectuate crop response

**Figure 11:**
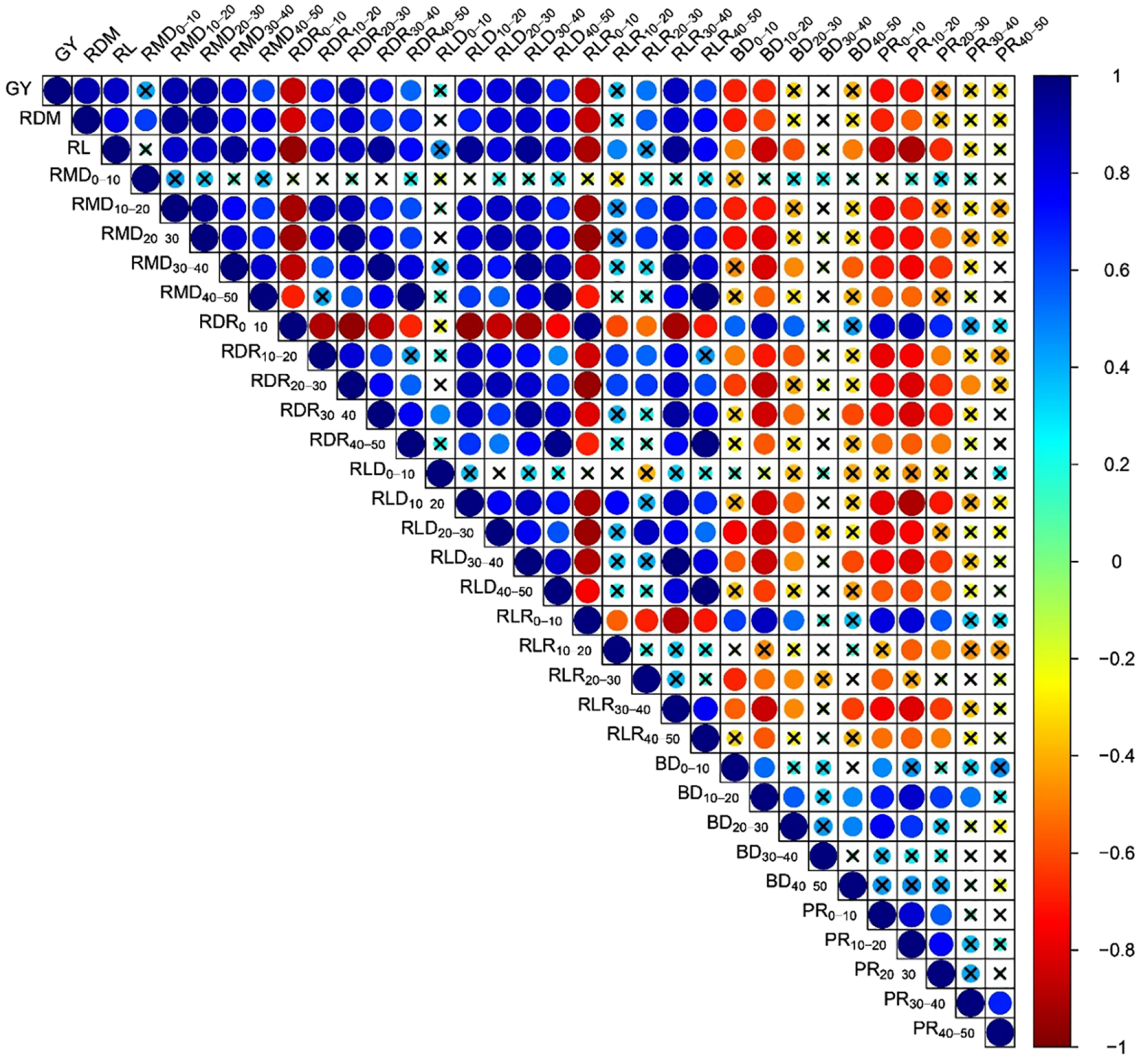
Correlation between grain yield, root distribution indices, and soil variables focusing soil depths in 2017. GY, grain yield; RDM, root dry matter per plant; RL, root length per plant; RMD, root mass density; RDR, root dry weight ratio; RLD, root length density; RLR, root length ratio; BD, bulk density; PR, penetration resistance; subscripts represent the corresponding soil depth where indicated. Circles represent correlation coefficients (r); and cross over circle shows insignificant correlation (p > 0.05).

As soil compaction has a more significant impact on crop root systems, most compaction-induced effects on crop performance originate from alterations in root growth, structure, and physiology (Martina et al., 2018). Results in this study show that the soil compaction not only reduced per plant root dry matter and length from but also effects were more noteworthy on root structure and distribution from 0 to 50 cm layers (**Figures 8**, **9** and **Tables 2**, **3**). Correlation analysis demonstrated a negative correlation of BD or PR (in 10-cm layers) with grain yield and root distribution variables (except for RDR and RLR in 0-10 cm layer), and these relationships were accentuated for BD and PR in the upper three 10-cm layers especially 10-20 cm layer (**Figure 11**). Compaction-induced alterations in BD and PR can hinder optimum root growth (Reichert et al., 2016), as revealed by significantly lower RMD and RLD in 10-40 cm soil profiles under trafficked plots. These results are in accordance with Mu et al. (2016) and Mosaddeghi et al. (2009), who found lower RMD and RLD due to higher soil BD and PR. Higher RDR and RLR in 0-10 cm soil layer under trafficked treatments showed that compaction promoted root growth in the surface layer. A higher concentration of roots in upper soil layers and reduced rooting in deeper layers can be due to the absence of larger diameter pores (Bengough et al., 2011; Lipiec et al., 2012) and resultantly in excessive mechanical impedance and insufficient oxygen supply in compacted soil layers (Nosalewicz and Lipiec, 2014). Moreover, increased horizontal cracks resulting from wheel trafficking might enhance superficial and horizontal root growth (Ball-Coelho et al., 1998). Even under control, >85% root dry matter and root length were distributed in the upper 30 cm layers, which signifies why topsoil compaction should be avoided even in the absence of subsoil compaction.

**Figure 12:**
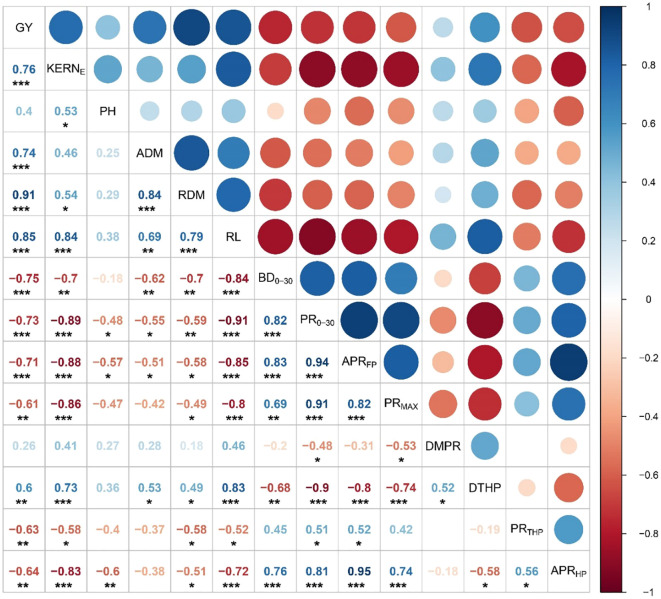
Correlation between selected crop variables, bulk density and penetration resistance in upper 30 cm soil profile, and penetration resistance indices in 2017. GY, grain yield; KERNE, kernels per ear; PH, plant height; ADM, aboveground dry matter per plant; RDM, root dry matter per plant; RL, root length per plant; BD0-30, bulk density in upper 30 cm soil; PR0-30, penetration resistance in upper 30 cm soil; APRFP, average PR up to 45 cm; PRMAX, maximum PR value; DMPR, depth to maximum PR value; DTHP, depth to the top of the hardpan layer; PRTHP, corresponding PR value at the top of the hardpan layer; APRHP, average PR in hardpan layer up to 45 cm. Circles and number represent correlation coefficients (r); and *, **, and *** show significant correlation at p < 0.05, p < 0.01, and p < 0.001, respectively.

**Figure 13:**
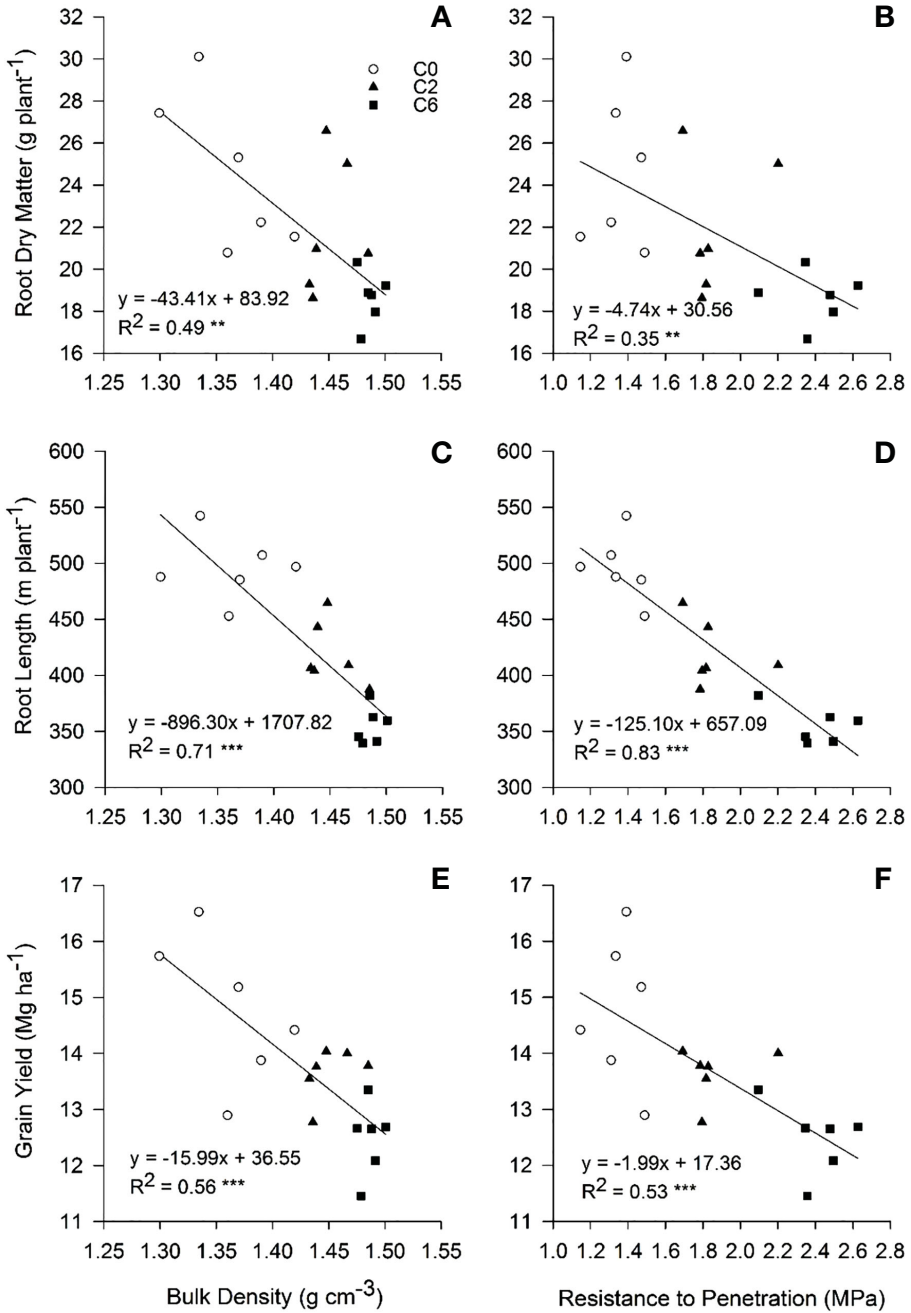
Relationship of root dry matter **(A, B)**, root length **(C, D)**, and grain yield **(E, F)** with soil bulk density and penetration resistance, respectively, in top soil (0-30 cm) under different compaction treatments in 2017. Treatments: C0, control; C2, two traffic passes; C6, six traffic passes. **, and *** show significant correlation at p <0.01, and p < 0.001, respectively.

When root growth is suppressed by soil compaction, shoot growth is bound to be affected (Montagu et al., 2001). BD and PR in topsoil (< 30 cm) and PR indices had significant relationships with various plant variables including grain yield (**Figure 12**). Root length was more strongly correlated to different PR indices than root dry matter. **Figure 13** shows significant negative relationships of root dry matter, root length, and grain yield with BD and PR in topsoil. Impaired air and water fluxes in compact soils can restrict root growth, blocking the ability of crops to explore and uptake the nutrient and water in the soil profile (Schäfer-Landefeld et al., 2004). However, mechanical impedance itself can negatively affect plant growth even if nutrients and water are not in limited supplies, as was evident from reduced leaf elongation under mechanically impeded root growth (Young et al., 1997; Bengough et al., 2006). Thus, we infer that traffic-induced alterations in soil properties affected root growth and distribution, affecting the absorption and utilization of soil nutrients and water by crops through their roots, and ultimately resulting in a negative response of overall crop growth and grain yield to field trafficking.

A previous study showed that the responses of the root architectures of maize cultivars to various soil compaction and moisture conditions were different (Xiong et al., 2020). In our study, it appeared from root distribution and visual observations (**Figures 9**, **10**) that XY had more root growth in deeper soil layers even under moderate soil compaction. Previously, researchers also concluded that XY had better root growth in deeper soil layers as compared with ZD (Zhang et al., 2013; Yu et al., 2015), which makes XY better suited under the condition of soil compaction.

## 5 Conclusions

Annual field trafficking with low axle weight machinery on relatively dry (moisture content lower than field capacity) sandy loam soil caused topsoil (<30 cm) compaction, however, no subsoil compaction or severe damage to soil structure was found. Most significant alterations in BD and PR were observed in 10-20 cm soil layer especially with the increase in vehicle passes. Field trafficking resulted in a shallower and stronger hardpan. Consequently, compaction restricted root proliferation in deeper layers of topsoil and promoted shallow horizontal root distribution resulting in reduced LAI and dry matter accumulation and partitioning; consequently, inflicting a grain yield penalty up to 15.5%. However, XY showed better grain yields than ZD under moderate compaction due to it deeper root distribution.

In crux, the current findings foreground traffic-induced soil compaction as a potential challenge for sustained crop production in the HHH region of China, where reduced or no tillage before summer maize plantation is a common practice. However, the magnitude of adverse effects of traffic-induced compaction on dry sandy loam soil in the current study was lesser than those involving heavier field traffic in wet field conditions, which suggests optimization of field traffic activity can avoid severe compaction.

## Data availability statement

The original contributions presented in the study are included in the article/supplementary material. Further inquiries can be directed to the corresponding authors.

## Author contributions

MMN: Performed, analyzed and wrote the manuscript MAN: analyzed and wrote the manuscript HL: Helped in data analysis XW: analyzed and wrote the manuscript WM: Conceived the idea and funded the project WZ: Conceived the idea. All authors contributed to the article and approved the submitted version.

## References

[B1] Arvidsson J. Håkansson I. (2014). Response of different crops to soil compaction–short-term effects in Swedish field experiments. Soil Tillage Res. 138, 56–63. doi: 10.1016/j.still.2013.12.006

[B2] Ball-Coelho B. R. Roy R. C. Swanton C. J. (1998). Tillage alters corn root distribution in coarse-textured soil. Soil Tillage Res. 45 (3), 237–249. doi: 10.1016/S0167-1987(97)00086-X

[B3] Batey T. (2009). Soil compaction and soil management – a review. Soil Use Manage. 25 (4), 335–345. doi: 10.1111/j.1475-2743.2009.00236.x

[B4] Bengough A. G. Bransby M. F. Hans J. McKenna S. J. Roberts T. J. Valentine T. A. (2006). Root responses to soil physical conditions; growth dynamics from field to cell. J. Exp. Bot. 57 (2), 437–447. doi: 10.1093/jxb/erj003 16317041

[B5] Bengough A. G. McKenzie B. M. Hallett P. D. Valentine T. A. (2011). Root elongation, water stress, and mechanical impedance: a review of limiting stresses and beneficial root tip traits. J. Exp. Bot. 62 (1), 59–68. doi: 10.1093/jxb/erq350 21118824

[B6] Berisso F. E. Schjønning P. Keller T. Lamandé M. Etana A. de Jonge L. W.. (2012). Persistent effects of subsoil compaction on pore size distribution and gas transport in a loamy soil. Soil Tillage Research, 122, 42–51. doi: 10.1016/j.still.2012.02.005

[B7] Blake G. R. Hartge K. H. (1986). “Bulk density,” in Methods of soil analysis: part 1–physical and mineralogical methods. Ed. Klute A. (Madison, WI: Soil Science Society of America, American Society of Agronomy), 363–375.

[B8] Blanco-Canqui H. Lal R. (2007). Regional assessment of soil compaction and structural properties under no-tillage farming. Soil Sci. Soc. America J. 71 (6), 1770–1778. doi: 10.2136/sssaj2007.0048

[B9] Botta G. F. Jorajuria D. Balbuena R. Rosatto H. (2004). Mechanical and cropping behavior of direct drilled soil under different traffic intensities: effect on soybean (Glycine max l.) yields. Soil Tillage Res. 78 (1), 53–58. doi: 10.1016/j.still.2004.01.004

[B10] Botta G. Jorajuria D. Draghi L. (1999). Soil compaction during secondary tillage traffic. Agro-Ciencia (Chile). 15, 139-144.

[B11] Botta G. F. Rivero D. Tourn M. Melcon F. B. Pozzolo O. Nardon G.. (2008). Soil compaction produced by tractor with radial and cross-ply tyres in two tillage regimes. Soil Tillage Res. 101 (1), 44–51. doi: 10.1016/j.still.2008.06.001

[B12] Botta G. F. Tolón-Becerra A. Rivero D. Laureda D. Ramírez-Roman M. Lastra-Bravo X.. (2016). Compactión produced by combine harvest traffic: effect on soil and soybean (Glycine max l.) yields under direct sowing in argentinean pampas. Eur. J. Agron. 74, 155–163. doi: 10.1016/j.eja.2015.12.011

[B13] Cai H. Ma W. Zhang X. Ping J. Yan X. Liu J.. (2014). Effect of subsoil tillage depth on nutrient accumulation, root distribution, and grain yield in spring maize. Crop J. 2 (5), 297–307. doi: 10.1016/j.cj.2014.04.006

[B14] Cambardella C. A. Elliott E. T. (1993). Carbon and nitrogen distribution in aggregates from cultivated and native grassland soils. Soil Sci. Soc. America J. 57 (4), 1071–1076. doi: 10.2136/sssaj1993.03615995005700040032x

[B15] Chamen W. C. T. Moxey A. P. Towers W. Balana B. Hallett P. D. (2015). Mitigating arable soil compaction: a review and analysis of available cost and benefit data. Soil Tillage Res. 146, 10–25. doi: 10.1016/j.still.2014.09.011

[B16] Chen G. Weil R. R. (2011). Root growth and yield of maize as affected by soil compaction and cover crops. Soil Tillage Res. 117, 17–27. doi: 10.1016/j.still.2011.08.001

[B17] Chen G. Weil R. R. Hill R. L. (2014). Effects of compaction and cover crops on soil least limiting water range and air permeability. Soil Tillage Res. 136, 61–69. doi: 10.1016/j.still.2013.09.004

[B18] da Silva A. P. Kay B. D. Perfect E. (1994). Characterization of the least limiting water range of soils. Soil Sci. Soc. America J. 58 (6), 1775–1781. doi: 10.2136/sssaj1994.03615995005800060028x

[B19] de Lima R. da Silva A. da Silva A. Leão T. Mosaddeghi M. (2016). Soilphysics: an r package for calculating soil water availability to plants by different soil physical indices. Comput. Electron. Agric. 120, 63–71. doi: 10.1016/j.compag.2015.11.003

[B20] de Lima R. P. da Silva A. P. Giarola N. F. B. da Silva A. R. Rolim M. M. (2017). Changes in soil compaction indicators in response to agricultural field traffic. Biosyst. Eng. 162, 1–10. doi: 10.1016/j.biosystemseng.2017.07.002

[B21] Duiker S. W. (2004a). Avoiding soil compaction. Pennsylvania State Univ. Extension Service 8.

[B22] Duiker S. W. (2004b). Effects of soil compaction. Pennsylvania State Univ. Extension Service 12.

[B23] Feike T. Doluschitz R. Chen Q. Graeff-Hönninger S. Claupein W. (2012). How to overcome the slow death of intercropping in the north China plain. Sustainability 4 (10), 2550–2565. doi: 10.3390/su4102550

[B24] Flint L. E. Flint A. L. (2002). “Porosity,” in Methods of soil analysis: part 4 physical methods. Eds. Dane J. H. Topp C. G. (Madison, WI: Soil Science Society of America), 241–254.

[B25] Gelder B. Cruse R. Zhang X. (2007). Comparison of track and tire effects of planter tractors on corn yield and soil properties. Trans. ASABE 50 (2), 365–370. doi: 10.13031/2013.22627

[B26] Gregorich E. G. Lapen D. R. Ma B. L. McLaughlin N. B. VandenBygaart A. J. (2011). Soil and crop response to varying levels of compaction, nitrogen fertilization, and clay content. Soil Sci. Soc. America J. 75 (4), 1483–1492. doi: 10.2136/sssaj2010.0395

[B27] Gregorich E. G. McLaughlin N. B. Lapen D. R. Ma B. L. Rochette P. (2014). Soil compaction, both an environmental and agronomic culprit: increased nitrous oxide emissions and reduced plant nitrogen uptake. Soil Sci. Soc. America J. 78 (6), 1913–1923. doi: 10.2136/sssaj2014.03.0117

[B28] Hallett P. Balana B. Towers W. Moxey A. Chamen T. (2012). Studies to inform policy development with respect to soil degradation. Sub project A: Cost curve for mitigation of soil compaction. Defra project SP1305.

[B29] Hamza M. A. Anderson W. K. (2005). Soil compaction in cropping systems: a review of the nature, causes and possible solutions. Soil Tillage Res. 82 (2), 121–145. doi: 10.1016/j.still.2004.08.009

[B30] Hartemink A. E. (2008). Soils are back on the global agenda. Soil Use Manage. 24 (4), 327–330. doi: 10.1111/j.1475-2743.2008.00187.x

[B31] Keller T. da Silva A. P. Tormena C. A. Giarola N. F. B. Cavalieri K. M. V. Stettler M.. (2015). SoilFlex-LLWR: linking a soil compaction model with the least limiting water range concept. Soil Use Manage. 31 (2), 321–329. doi: 10.1111/sum.12175

[B32] Kemper W. D. Rosenau R. C. (1986). “Aggregate stability and size distribution,” in Methods of soil analysis: part 1–physical and mineralogical methods. Ed. Klute A. (Madison, WI: Soil Science Society of America, American Society of Agronomy), 425–442.

[B33] Lipiec J. Hatano R. (2003). Quantification of compaction effects on soil physical properties and crop growth. Geoderma 116 (1-2), 107–136. doi: 10.1016/s0016-7061(03)00097-1

[B34] Lipiec J. Horn R. Pietrusiewicz J. Siczek A. (2012). Effects of soil compaction on root elongation and anatomy of different cereal plant species. Soil Tillage Res. 121, 74–81. doi: 10.1016/j.still.2012.01.013

[B35] Liu Z. Zhu K. Dong S. Liu P. Zhao B. Zhang J. (2017). Effects of integrated agronomic practices management on root growth and development of summer maize. Eur. J. Agron. 84, 140–151. doi: 10.1016/j.eja.2016.12.006

[B36] Lunne T. Robertson P. K. Powell J. J. M. (1997). Cone penetration testing in geotechnical practice (UK: Blackie Academic. Chapman and Hall Publishers).

[B37] Martina C. Barbara M. Fabio F. Alberto M. Andrea T. Cristiano F.. (2018). Early response of quercus robur seedlings to soil compaction following germination. Land Degradation Dev. 29 (4), 916–925. doi: 10.1002/ldr.2912

[B38] Meng E. C. H. Hu R. Shi X. Zhang S. (2006). Maize in China: production systems, constraints, and research priorities (Mexico, D.F: CIMMYT).

[B39] Mirleau-Thebaud V. Dayde J. Scheiner J. D. (2017). Growth kinetics at early stages of sunflower (Helianthus annuus l.) under soil compaction. J. Plant Nutr. 40 (17), 2494–2510. doi: 10.1080/01904167.2017.1380820

[B40] Montagu K. D. Conroy J. P. Atwell B. J. (2001). The position of localized soil compaction determines root and subsequent shoot growth responses. J. Exp. Bot. 52 (364), 2127–2133. doi: 10.1093/jexbot/52.364.2127 11604451

[B41] Montgomery E. (1911). Correlation studies in corn. Annu. Rep. No. 24 Nebraska Agric. Experiment Station 24, 108–159).

[B42] Mosaddeghi M. R. Mahboubi A. A. Safadoust A. (2009). Short-term effects of tillage and manure on some soil physical properties and maize root growth in a sandy loam soil in western Iran. Soil Tillage Res. 104 (1), 173–179. doi: 10.1016/j.still.2008.10.011

[B43] Mu X. Zhao Y. Liu K. Ji B. Guo H. Xue Z.. (2016). Responses of soil properties, root growth and crop yield to tillage and crop residue management in a wheat–maize cropping system on the north China plain. Eur. J. Agron. 78, 32–43. doi: 10.1016/j.eja.2016.04.010

[B44] Nimmo J. R. Perkins K. S. (2002). “Aggregate stability and size distribution,” in Methods of soil analysis: part 4 physical methods. Eds. Dane J. H. Topp C. G. (Madison, WI: Soil Science Society of America), 317–328.

[B45] Nosalewicz A. Lipiec J. (2014). The effect of compacted soil layers on vertical root distribution and water uptake by wheat. Plant Soil 375 (1), 229–240. doi: 10.1007/s11104-013-1961-0

[B46] Osaki M. Shinano T. Matsumoto M. Ushiki J. Shinano M. M. Urayama M.. (1995). Productivity of high-yielding crops. Soil Sci. Plant Nutr. 41 (4), 635–647. doi: 10.1080/00380768.1995.10417014

[B47] Reichert J. M. Rodrigues M. F. Bervald C. M. P. Kato O. R. (2016). Fire-free fallow management by mechanized chopping of biomass for sustainable agriculture in Eastern Amazon: effects on soil compactness, porosity, and water retention and availability. Land Degradation Dev. 27 (5), 1403–1412. doi: 10.1002/ldr.2395

[B48] Schäfer-Landefeld L. Brandhuber R. Fenner S. Koch H.-J. Stockfisch N. (2004). Effects of agricultural machinery with high axle load on soil properties of normally managed fields. Soil Tillage Res. 75 (1), 75–86. doi: 10.1016/S0167-1987(03)00154-5

[B49] Schjønning P. Lamandé M. Munkholm L. J. Lyngvig H. S. Nielsen J. A. (2016b). Soil precompression stress, penetration resistance and crop yields in relation to differently-trafficked, temperate-region sandy loam soils. Soil Tillage Res. 163, 298–308. doi: 10.1016/j.still.2016.07.003

[B50] Schjønning P. van den Akker J. J. H. Keller T. Greve M. H. Lamandé M. Simojoki A.. (2016a). “Soil compaction,” in Soil threats in Europe - EUR 27607 EN. Luxembourg: joint research centre, the European commission. Eds. Stolte J. Tesfai M. Øygarden L. Kværnø S. Keizer J. Verheijen F. Panagos P. Ballabio C. Hessel R.. (Luxembourg:EU joint research centre).

[B51] Shah A. N. Tanveer M. Shahzad B. Yang G. Fahad S. Ali S.. (2017). Soil compaction effects on soil health and cropproductivity: an overview. Environ. Sci. pollut. Res. 24 (11), 10056–10067. doi: 10.1007/s11356-017-8421-y 28108925

[B52] Shen P. Wu Z. Wang C. Luo S. Zheng Y. Yu T.. (2016). Contributions of rational soil tillage to compaction stress in main peanut producing areas of China. Sci. Rep. 6, 38629. doi: 10.1038/srep38629 27934905 PMC5146654

[B53] Sidhu D. Duiker S. W. (2006). Soil compaction in conservation tillage. Agron. J. 98 (5), 1257–1264. doi: 10.2134/agronj2006.0070

[B54] Sivarajan S. Maharlooei M. Bajwa S. G. Nowatzki J. (2018). Impact of soil compaction due to wheel traffic on corn and soybean growth, development and yield. Soil Tillage Res. 175, 234–243. doi: 10.1016/j.still.2017.09.001

[B55] Soane B. D. Ouwerkerk C. V. (1994). Soil compaction in crop production (Amsterdam, Netherlands: Elsevier).

[B56] Sweeney D. W. Kirkham M. B. Sisson J. B. (2006). Crop and soil response to wheel-track compaction of a claypan soil. Agron. J. 98 (3), 637–643. doi: 10.2134/agronj2005.0254

[B57] Tekeste M. Z. Raper R. L. Schwab E. B. (2008). Soil drying effects on soil strength and depth of hardpan layers as determined from cone index data. Agric. Eng. International: CIGR J. 10.

[B58] Tolon-Becerra A. Tourn M. Botta G. F. Lastra-Bravo X. (2011). Effects of different tillage regimes on soil compaction, maize (Zea mays l.) seedling emergence and yields in the eastern argentinean pampas region. Soil Tillage Res. 117, 184–190. doi: 10.1016/j.still.2011.10.003

[B59] Tubeileh A. Groleau-Renaud V. Plantureux S. Guckert A. (2003). Effect of soil compaction on photosynthesis and carbon partitioning within a maize–soil system. Soil Tillage Res. 71 (2), 151–161. doi: 10.1016/S0167-1987(03)00061-8

[B60] Voorhees W. (2000). Long-term effect of subsoil compaction on yield of maize. Adv. GeoEcology 32, 331–338.

[B61] Wolkowski R. P. (1990). Relationship between wheel-Traffic-Induced soil compaction, nutrient availability, and crop growth: a review. J. Production Agric. 3 (4), 460–469. doi: 10.2134/jpa1990.0460

[B62] Xiong P. Zhang Z. Hallett D. P. Peng X. (2020). Variable responses ofmaize root architecture in elite cultivars due to soil compaction and moisture. Plant Soil 455, 79–91.

[B63] Young I. M. Montagu K. Conroy J. Bengough A. G. (1997). Mechanical impedance of root growth directly reduces leaf elongation rates of cereals. New Phytol. 135 (4), 613–619. doi: 10.1046/j.1469-8137.1997.00693.x

[B64] Yu P. Li X. White P. J. Li C. (2015). A Large and deep root system underlies high nitrogen-use efficiency in maize production. PloS One 10 (5), e0126293. doi: 10.1371/journal.pone.0126293 25978356 PMC4433229

[B65] Zhai L. Xu P. Zhang Z. Li S. Xie R. Zhai L.. (2017). Effects of deep vertical rotary tillage on dry matter accumulation and grain yield of summer maize in the Huang-Huai-Hai plain of China. Soil Tillage Res. 170, 167–174. doi: 10.1016/j.still.2017.03.013

[B66] Zhang X. Y. Cruse R. M. Sui Y. Y. Jhao Z. (2006). Soil compaction induced by small tractor traffic in northeast China. Soil Sci. Soc. America J. 70 (2), 613–619. doi: 10.2136/sssaj2005.0121

[B67] Zhang F. L. Niu X. K. Zhang Y. M. Xie R. Z. Liu X. Li S. K.. (2013). Studies on the root characteristics of maize varieties of different eras. J. Integr. Agric. 12 (3), 426–435. doi: 10.1016/S2095-3119(13)60243-9

